# RNA helicase p68 deploys β-catenin in regulating RelA/p65 gene expression: implications in colon cancer

**DOI:** 10.1186/s13046-019-1304-y

**Published:** 2019-07-27

**Authors:** Veenita Khare, Shaheda Tabassum, Uttara Chatterjee, Sandip Chatterjee, Mrinal K. Ghosh

**Affiliations:** 1 Cancer Biology and Inflammatory Disorder Division, Council of Scientific and Industrial Research-Indian Institute of Chemical Biology (CSIR-IICB), TRUE Campus, CN-6, Sector-V, Salt Lake, Kolkata- 700091 & 4, Raja S.C. Mullick Road, Jadavpur, Kolkata, 700032 India; 2 Division of Pathology, Park Clinic, 4, Gorky Terrace, Kolkata, 700017 India

**Keywords:** Colorectal cancer, RNA helicase p68, Wnt/β-catenin signaling, NF-κB signaling cascade, RelA gene regulation, Murine model

## Abstract

**Background:**

RelA/p65 a crucial member of NF-κB signaling pathway plays diverse role in mediating oncogenesis. Limited knowledge prevails on the mechanistic insights of RelA gene regulation. RNA helicase p68 apart from being a vital player of RNA metabolism acts as a transcriptional coactivator of several oncogenic transcription factors including β-catenin and is highly implicated in cancer progression. In this study, we aim to discern the molecular mechanism of how an RNA helicase, p68 deploys a major oncogenic signaling pathway, Wnt/ β-catenin to regulate the expression of RelA, an indispensable component of NF-κB signaling pathway towards driving colon carcinogenesis.

**Methods:**

Immunoblotting and quantitative RT-PCR was performed for determining the protein and mRNA expressions of the concerned genes respectively. Luciferase assay was employed for studying the promoter activity of RelA. Chromatin immunoprecipitation was used to evaluate the occupancy of transcription factors on the RelA promoter. Immunohistochemical analysis was conducted using FFPE sections derived from normal human colon and colon cancer patient samples. Finally, a syngeneic colorectal allograft mouse model was used to assess physiological significance of the *in vitro* findings.

**Results:**

p68, β-catenin and RelA proteins were found to bear strong positive correlation in normal and colon carcinoma patient samples. Both p68 and β-catenin increased RelA mRNA and protein expression. p68, β-catenin and Wnt3a elevated RelA promoter activity. Conversely, p68 and β-catenin knockdown diminished RelA promoter activity and led to reduced RelA mRNA and protein expression. p68 was perceived to occupy RelA promoter with β-catenin at the TCF4/LEF (TBE) sites thereby potentiating RelA transcription. p68 and β-catenin alliance positively modulated the expression of signature NF-κB target genes. Enhanced NF-κB target gene expression by p68 was corroborated by findings in clinical samples. Tumors generated in mice colorectal allograft model, stably expressing p68 further reinforced our *in vitro* findings.

**Conclusions:**

We report for the first time a novel mechanism of alliance between p68 and β-catenin in regulating the expression of RelA and stimulating the NF-κB signaling axis towards driving colon carcinogenesis. This study unravels novel modes of p68-mediated colon carcinogenesis, marking it a potential target for therapy.

**Electronic supplementary material:**

The online version of this article (10.1186/s13046-019-1304-y) contains supplementary material, which is available to authorized users.

## Background

p68/DDX5 is a prototypical member of the DEAD box family of RNA helicases and participates in nearly all aspects of RNA metabolism. Mounting evidences suggest p68 to play a crucial role in cancer development and progression. It is reported to be overexpressed in various cancers and acts as a transcriptional co-activator of several transcription factors like β-catenin, NF-κB p50, MAML1 and Androgen Receptor [1–4].

In terms of both morbidity and mortality, colorectal cancer (CRC) is one of the most common adult malignant tumors worldwide. Aberrant regulation of Wnt/β-catenin signaling cascade is the prevalent theme in colon carcinogenesis, β-catenin being the central oncogenic driver. In CRC, mutations in APC, β-catenin gene or up-regulation of Wnt pathway disrupts the cytoplasmic destruction complex mediated regulation of β-catenin. This enables β-catenin to accumulate in the cytoplasm, translocate to the nucleus and form complex with TCF/LEF HMG box transcription factors including TCF4. This interaction ensures efficient activation of specific target genes that drives initial as well as later stages of neoplastic transformation and progression [5].

Abnormal inflammatory response plays a critical role in colon carcinogenesis. Nuclear factor kappa B (NF-κB) activation, a cardinal feature of tumorigenesis has been proposed to act as a crucial link between inflammation and cancer. Mammalian NF-κB is a family of ubiquitous transcription factors and consists of five members, sharing a highly conserved Rel homology domain (RHD): Rel A (p65), c-Rel, Rel B, NF-κB1 (p50) and NF-κB2 (p52). These factors exist in heterodimeric or homodimeric complexes of which heterodimer p65/p50 is the most predominant. In unstimulated cells, NF-κB complexes are sequestered in the cytoplasm through their interaction with members of the inhibitor of κB (IκB) family. Specific cellular stimulation activates IκB kinase (IKK) complex which phosphorylates IκB leading to its ubiquitination and proteosome-mediated degradation. Following IκB degradation, NF-κB translocates to the nucleus to promote the transcription of various target genes bearing consensus κB sequences in the promoter or enhancer regions [6, 7].

The pivotal role played by NF-κB in mediating colon carcinogenesis is illustrated by its regulation of a great diversity of genes positioned at the interface between cell proliferation, survival and evasion from apoptosis. Several evidences support the role of NF-κB in regulating the expression of antiapoptotic members of the Bcl-2 family like Bcl-2 [8] and Bcl-xL [9]. Member of the IAP gene family XIAP [10] and Survivin [11] have been identified as downstream targets of NF-κB signaling pathway. Overexpression of these genes are associated with tumor development, progression, metastasis and chemo- or radio-resistance thus making them good biomarkers for determining aggressiveness of colorectal adenocarcinomas [12, 13].

Regulation of NF-κB activity occurs at multiple levels [14]; hence it is not simply adequate to understand the mechanism of nuclear localization and DNA binding of NF-κB subunits. Comprehending the molecular mechanism behind the regulation of each subunit of NF-κB complex and determination of their functionality will help to elucidate the NF-κB pathway as a whole. Limited knowledge exists regarding the transcriptional regulation of the NF-κB family member RelA/p65. RelA is reported to be overexpressed in several cancers including colon and patients with high RelA expression exhibit poor prognosis [15]. Studies have reported Sp1 to play a key role in transactivation of the RelA gene [16, 17].

A lot remains to be explored regarding the mechanisms of RelA gene regulation, an indispensable member of NF-κB signaling pathway. p68 serves as a coactivator of β-catenin [4] and β-catenin has been observed to act upon inflammatory processes, often via crosstalk with NF-κB pathway, but the mechanistic details behind it largely remains unknown [18]. It was intriguing to investigate the involvement of p68 as a coactivator of β-catenin in RelA gene regulation and to study the subsequent effect of this synergism on NF-κB signaling axis. Understanding the molecular mechanism that regulates RelA function will pave way for elucidating the role NF-κB subunits play in human inflammatory diseases and cancer, and bear a strong impact on the use of future NF-κB based cancer therapies. Thus, from this study we could infer p68 being the intersecting point between Wnt/β-catenin and NF-κB signaling pathway, the two major signaling cascades that drive CRC.

## Methods

### Human colon tissue samples

Formalin-fixed paraffin-embedded (FFPE) post-surgical human normal (*n* = 22) colon and colon carcinoma (*n* = 45) samples were collected from Indian patients. All clinical and ethical regulations were followed for collecting the samples, including patient consent and with formal approval from the ethical committee of both CSIR-IICB and Park Clinic (source) [19].

### Cell culture and transfection

Human colorectal cancer (CRC) cell lines [HCT 116, SW-480, HT-29, HCT-15]; human embryonic kidney cell line [HEK-293] and murine adenocarcinoma cells [CT26] were procured from ATCC (Manassas, VA, USA). The cell lines were cultured in McCoy’s 5A, DMEM or RPMI-1640, as required, supplemented with 10% heat-inactivated fetal bovine serum (FBS) and maintained at 5% CO2, 37 °C in a humid incubator. Penicillin/Streptomycin cocktail and Gentamycin (Invitrogen, Life Technologies) were added as prescribed antibiotic at the recommended dose. Transfections of different DNA constructs were performed using Lipofectamine 2000 (Invitrogen). Lipofectamine RNAi MAX (Invitrogen) was used for siRNA-mediated knockdown experiments. Small interfering RNAs (siRNAs) against β-catenin, p68 and TCF4 were purchased from Santa Cruz Biotechnology (Santa Cruz, CA, USA). CT26 cells were transfected using Lipofectamine LTX (Life Technologies), G418 (Calbiochem, Darmstadt, Germany) (500 μg/ml) was used for selection of stable cell line.

### Expression plasmid constructs and cloning

p68 was sub-cloned from pSG5-Myc vector into pGZ21dx, pEGFP-c3 and pIRES-hrGFP-1a. pBI-β-catenin was sub-cloned into pGZ21dx vector and human Wnt3a gene was cloned in pcDNA3.1-myc-his, respectively. RelA promoter region (1746 bp fragment) was PCR amplified using genomic DNA from HEK-293 cells and following standard protocol cloned into pGL3 basic vector. ALGGEN-PROMO (http://alggen.lsi.upc.es/) was used for putative analysis of the transcription factors binding to this region. All the constructs were verified by restriction digestion and confirmed by sequencing. All primers were synthesized from IDT (Integrated DNA Technologies, Coralville, IA, USA). Sequences of the primers are given in Additional file 5: Table S1.

### Site-directed mutagenesis

Deletion of TBE consensus sites (CTTTG [sites 2,4,6,7]/CAAAG [sites 1,3,5]) in the RelA promoter construct were performed using the QuickChange XL Site-Directed Mutagenesis kit (Agilent Technologies, Santa Clara, CA, USA), following manufacturer’s instructions. All deletion constructs were verified by DNA sequencing. Sequences of the primers are given in Additional file 5: Table S1.

### RNA interference mediated knockdown

Two different shRNAs targeting p68 and a control (scrambled) shRNA were subcloned in pMKO.1-Puro according to Addgene’s protocol. shRNA targeting β-catenin was purchased from Addgene (pLKO.1 puro shRNA β-catenin) (Addgene Cat no 18803). Scramble siRNA, p68, β-catenin and TCF4 siRNA (Santa Cruz Biotechnology, Inc., Dallas, TX, USA) were used at a final concentration of 30 nM.

### Immunoblot analysis

Preparation of whole cell lysates (WCL), cytoplasmic and nuclear extracts and immunoblot analyses were performed as described previously [20, 21]. The following primary antibodies were used: p68 (Abcam); Cyclin D1, RelA/p65, Bcl-2, XIAP, Bcl-xL, Survivin, TCF4 (Cell Signaling Technology); α-Tubulin, Lamin B, Actin, GFP, β-catenin and GAPDH (Santacruz Biotechnology); HRP-tagged anti-rabbit and anti-mouse secondary antibodies (Cell Signaling Technology); HRP-tagged anti-goat secondary antibody (Sigma-Aldrich). GelQuant. Net software (http://biochemlabsolutions.com/GelQuantNET.html) was used for densitometric scanning of immunoblots. Blots were evaluated by using Luminata Classico Western HRP Substrate (Millipore) following manufacturer’s protocol.

### Quantitative real-time PCR

Total RNA was extracted using the Trizol reagent (Invitrogen, NY, USA), converted to cDNA (1 μg of RNA for each sample) using RevertAid H Minus First Strand cDNA Synthesis Kit (Thermo Scientific™) which was subsequently used for qRT-PCR analysis using FastStart Universal SYBR Green Master (Roche) on a ViiA 7 Real-Time PCR system (Applied Biosystems). In all the experiments, 18S rRNA served as the internal control. Quantification and Standard deviation (SD) calculations were based on three independent experiments in triplicates. Sequences of the primers are given in Additional file 5: Table S1.

### Luciferase based promoter activity assay

Cells were transiently transfected with pGL3-RelA promoter luciferase reporter construct along with Renilla luciferase plasmid (pRL-TK) and respective gene constructs as per experimental interest and indicated in the relevant figure(s). Luciferase Assay was performed with the cell lysates 36 or 48 h post-transfection following the manufacturer’s protocol. The efficiency of transfection was normalized with Renilla luciferase expression. Quantification was based on three independent experiments. Luciferase activity was determined luminometrically in the Varioskan Flash Multimode Reader (Thermo Fisher Scientific, Waltham, MA, USA) by the dual luciferase assay system (Promega, Madison, WI, USA) following manufacturer’s instructions and as described previously [22].

### ChIP (chromatin immunoprecipitation) assay

The preparation of chromatin and the immunoprecipitation of such for ChIP assay were performed as described earlier [23, 24]. ChIP Quantitative RT-PCR (qRT-PCR) reactions were performed using SYBR Green master mix. Sequences of the primers are given in Additional file 5: Table S1.

### Tissue histology and immunohistochemical (IHC) analysis

For histology and IHC analysis, 5 μm thick sections from both human patient samples and murine allograft tumors were prepared from paraffin-embedded blocks. For histological analysis, the tissue sections were subjected to hematoxylin and eosin (H&E) staining following standard protocol. IHC was performed using SuperSensitive™ Polymer-HRP IHC Detection System (BioGenex), following manufacturer’s instructions and as described previously [19]. Images were captured using EVOS XL microscope (Life technologies) at 200X optical magnifications. A semiquantitative scoring method was employed for assessing the intensity of staining and generation of H-score which was used for further statistical analysis. Mayer’s Hematoxylin (#MHS1; Sigma, St. Louis, MO, USA) was used as a counter stain for nucleus. The following primary antibodies were used: p68, Snail (Abcam); RelA, TCF4, PCNA, Bcl-2, Bcl-xL, XIAP, Survivin and Vimentin (Cell Signaling Technology); β-catenin and Bcl-2 (Santacruz Biotechnology).

### Murine colorectal allograft tumor model

All animal care and experimentation were conducted according to the CPCSEA guidelines; with approval of the animal ethics committee at CSIR-Indian Institute of Chemical Biology. Murine adenocarcinoma cell line CT26 was used for generation of syngeneic tumor model.

#### Primary tumor growth

To generate the primary tumor model, BALB/c mice (*n* = 6; 4–5 weeks old; 18–20 g) were injected with 10^6^ CT26 cells stably expressing EGFP or EGFP-p68, subcutaneously in the right flank region. The onset of tumor development in the animals was monitored daily by palpating the injection area. Growth of the tumor was observed for a period of 30 days after which the mice were sacrificed and the tumor was excised. The tumor volume was measured by vernier calipers. The tumor volume was determined as [length × width^2^] × 0.5. Tumors were fixed in 10% buffered formalin and embedded in paraffin for histology and immunohistochemical (IHC) analysis.

#### Metastatic tumor growth

5 × 10^5^ CT26 cells stably expressing EGFP or EGFP-p68 were injected into the tail vein of BALB/c mice (*n* = 6; 4–5 weeks old; 18–20 g) to generate metastatic growth in lung. 30 days post injection, animals were sacrificed, the lungs were removed, metastatic nodules were counted and the diameter of the nodules was measured. Lungs were fixed in 10% buffered formalin and embedded in paraffin for further processing.

### Statistical analysis

At least three independent experiments were performed in triplicates to calculate means and standard deviations (SD). To determine the significance values in all the experiments, Student’s t-test (unpaired) was performed, using GraphPad QuickCals (http://www.graphpad.com/quickcalcs/index.cfm). H-scores were calculated manually. Mann–Whitney U-test was used to evaluate the differences in H-score values of all the concerned proteins between human normal colon and colon carcinoma samples, using R module Wilcoxon-Mann-Whitney Test calculator (https://www.wessa.net/rwasp_Reddy-Moores%20Wilcoxon%20Mann-Witney%20Test.wasp). Distribution of H-scores are represented by Box plots, generated using web tool BoxPlotR (http://boxplot.tyerslab.com/). Spearman’s rank correlation coefficient (r_s_) was used to determine the correlation between the expression levels of two different proteins (individual H-scores) in both human normal colon and colon carcinoma samples, using R module Spearman Rank Correlation calculator (http://www.wessa.net/rwasp_spearman.wasp/). Throughout, the value of *P* < 0.05 was considered to be statistically significant (*), *P* < 0.01, *P* < 0.001 and *P* < 0.0001 were considered to be very significant (**), highly significant (***) and extremely significant (****), respectively.

## Results

### p68 and RelA maintain positive correlation in colon carcinoma tissue samples and cell lines indicating p68 may regulate RelA protein expression

**Fig. 1:**
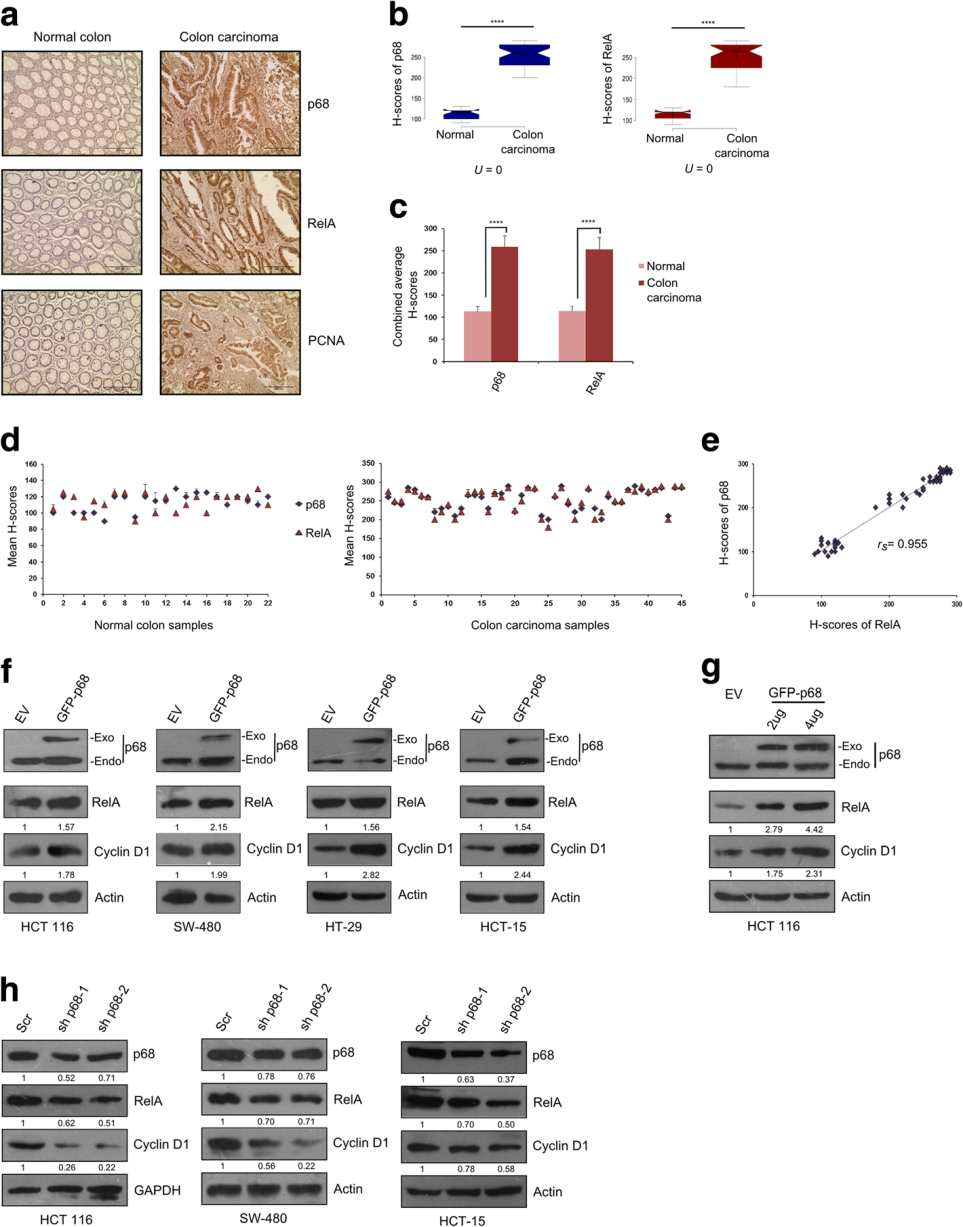
p68 and RelA abundance is positively correlated and RelA protein expression is regulated by p68 in human colon cancer. **a** Representative images depicting immunohistochemical (IHC) staining of p68, RelA and PCNA conducted in tissue sections derived from human normal colon and colon carcinoma samples. Images were captured at 200X magnification. **b** Notched box plots showing the distribution of H-scores of p68 and RelA in normal (*n* = 22) and colon carcinoma (*n* = 45) samples; Mann–Whitney U-values were calculated from H-scores. **c** Comparison of the combined average H-scores of p68 and RelA. Error bars represent the mean (+) s.d.; *P* < 0.0001 is represented as ****; calculated using Student’s t-test. **d** Scatter plots representing the mean H-scores of p68 and RelA in normal and colon carcinoma tissue samples, respectively. **e** Determination of Spearman’s rank correlation coefficient (rs) between the mean H-scores of p68 and RelA, analyzed from both normal and colon carcinoma tissues in combination. **f** Multiple colon cancer cell lines as depicted in figure, grown in 60 mm cell culture dishes were transfected with 4 μg of pGZ-p68 or the empty vector (EV). **g** HCT 116 cells were seeded in 60 mm plates and were transfected with EV or pGZ-p68 in a dose-dependent manner as indicated. For both (**f**) and (**g**) cell lysates were prepared, 36 h post transfection and probed for the indicated proteins by immunoblotting (IB). ‘Exo’ and ‘Endo’ depicts exogenous and endogenous p68, respectively. Densitometry values below the blots in both panels (**f**) and (**g**) are relative to the loading control (Actin). **h** Multiple colon cancer cell lines were seeded in 60 mm cell culture dishes and transfected with 4 μg of either scramble shRNA (represented as scr), p68 shRNA1 or p68 shRNA2 (represented as sh p68–1 and sh p68–2 respectively) plasmids. Cell lysates were prepared, 48 h post transfection and probed for the indicated proteins by IB. Densitometry values relative to loading control (GAPDH/Actin) are given below the blots

p68 is reported to be overexpressed in colon cancer samples, implicating it as a favorable prognostic marker for colon carcinoma [1, 2]. Increased RelA expression in colon carcinoma samples has been substantiated by several studies [15]. To investigate the possible connection of p68 with RelA, we conducted immunohistochemical (IHC) analysis in normal and colon carcinoma tissue samples procured from Indian patients. The expression of p68 and RelA exhibited marked elevation in colon carcinoma samples compared to that of normal. Expression of PCNA served as a proliferation marker (Fig. 1a). The difference in staining intensities (measured by H-score) of p68 and RelA between normal and carcinoma samples (represented by Notched Box Plot) were statistically significant, as predicted by the Mann–Whitney U-test analysis (Fig. 1b). Also, the difference between the average H-scores of p68 and RelA in colon carcinoma samples was statistically significant compared to the normal samples (Fig. 1c). Staining intensities of p68 and RelA (measured by H-score) followed similar trends in both normal and carcinoma samples (Fig. 1d). H-scores of p68 and RelA was found to bear strong positive correlation (rs = 0.955), when statistically analyzed by Spearman’s rank correlation test (Fig. 1e). These results indicate conceivable interconnection between p68 and RelA.

To investigate whether p68 regulates RelA gene expression, p68 was overexpressed in multiple colorectal cancer (CRC) cell lines. In all the cases, p68 enhanced RelA protein expression. p68 co-activates β-catenin in regulating its target gene Cyclin D1 [4]. Hence, Cyclin D1 served as positive control for p68 overexpression, which also displayed similar trends of expression like RelA (Fig. 1f). To further delve into the possible effect of p68 on RelA, we overexpressed p68 in a dose-dependent manner in HCT 116 cells. The results demonstrated dose-dependent increment in the expression of RelA, and also that of Cyclin D1 following a similar course (Fig. 1g). Next, in a converse approach, we used multiple short hairpin RNA (shRNA) against p68 to deplete its endogenous level. Significant reduction in RelA expression was observed on knocking down p68. Cyclin D1 followed a corresponding trend (Fig. 1h). Altogether, these results indicate highly plausible interconnection between p68 and RelA in both clinical samples and CRC cell lines and reinforces our hypothesis of p68 being involved in regulating RelA gene expression.

### p68 regulates RelA gene expression by occupying its promoter at the TCF-LEF binding elements (TBEs)

**Fig. 2:**
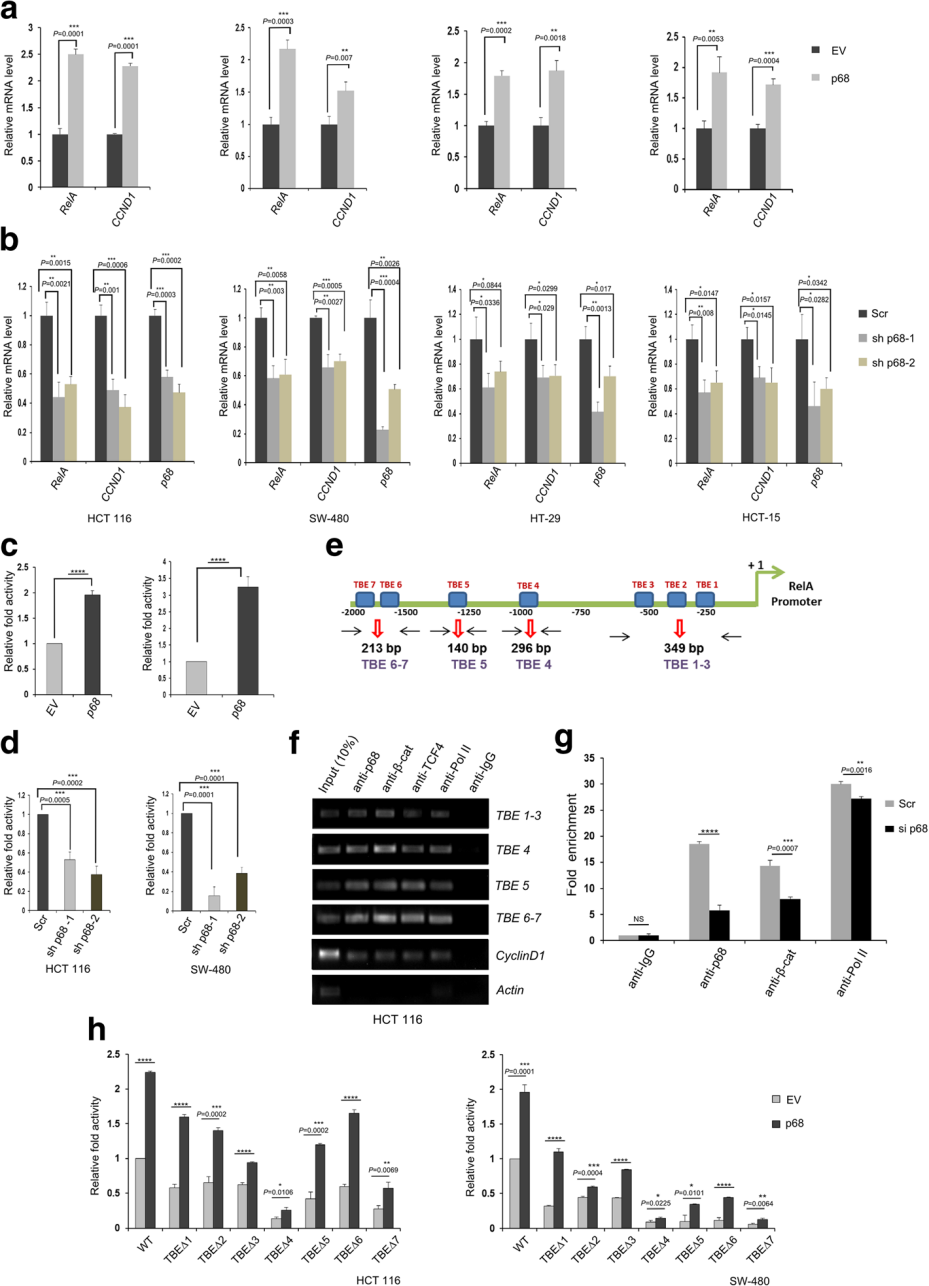
p68 transcriptionally regulates RelA gene expression through its occupancy at the TBE sites of the RelA promoter. **a** Multiple colon cancer cell lines as indicated were seeded in 35 mm plates and transfected with 2 μg of pGZ-p68 or EV. Total RNA was extracted 36 h post transfection followed by analysis of RelA mRNA by qRT–PCR. **b** Multiple colon cancer cell lines as indicated were seeded in 35 mm plates and transfected with 2 μg of scr (control), or sh p68–1 or sh p68–2. Total RNA was extracted 48 h post transfection, followed by analysis of RelA mRNA by qRT–PCR. For both (**a**) and (**b**) Cyclin D1 (CCND1) mRNA expression served as positive control. 18S rRNA was used for normalization. **c** HCT 116 (left) and SW-480 (right) cells were seeded in 35 mm plates and co-transfected with pGL3-RelA-prom (1 μg), Renilla luciferase plasmid (50 ng) and either EV or pIRES-p68 (1 μg). The luciferase activity was measured, 36 h post transfection. **d** HCT 116 (left) and SW-480 (right) cells were seeded in 35 mm plates and transfected with either shRNA1 (1 μg) or shRNA2 (1 μg) of p68 or scr (control- EV) (1 μg) along with pGL3-RelA-prom (1 μg) and Renilla luciferase plasmid (50 ng). The luciferase activity was measured, 48 h post transfection. For both (**c**) and (**d**) cells co-transfected with 1 μg of EV, pGL3-RelA-prom and Renilla luciferase plasmid (50 ng) served as control. **e** Schematic representation of the RelA promoter showing multiple TCF-binding elements (TBE) sites. **f** HCT 116 cells were grown in 100 mm cell culture dishes followed by Chromatin immunoprecipitation (ChIP) assay using the indicated antibodies. Amplification of Cyclin D1 promoter region containing TBE served as positive control for both p68 and β-catenin. Actin promoter served as positive control for Pol II. PCR amplification of the immunoprecipitated DNAs was performed using primers designed from the RelA promoter region – TBE [1–3]-(349 bp), flanking TBE1, TBE2 and TBE3; TBE4-(296 bp), flanking TBE4; TBE5-(140 bp), flanking TBE5 and TBE [6–7]-(213 bp), flanking TBE6 & TBE7. **g** HCT 116 cells were grown in 100 mm plates and transfected with 8 μl scr (control) or siRNA against p68 (si p68) followed by ChIP assay using the indicated antibodies; 48 h post transfection. Quantification of the immunoprecipitated DNA was done by qRT–PCR. Results were determined first as percentage of input and then presented as fold enrichment relative to IgG. For both (**f**) and (**g**) ChIP using antibody against RNA Polymerase II (Pol II) and IgG served as positive and negative control, respectively. DNA extract (10% without ChIP) was used as input. NS denotes ‘not significant’. **h** HCT 116 (left) and SW-480 (right) cells seeded in 35 mm plates were transfected with either pIRES-p68 or pIRES-EV (1 μg) along with pGL3-WT-RelA-prom or its deletion constructs (1 μg) and Renilla luciferase plasmid (50 ng). Cells co-transfected with (1 μg) of EV (pIRES), pGL3-WT-RelA-prom and Renilla luciferase plasmid (50 ng) served as control. The luciferase activity was measured, 36 h post transfection. For (**c**), (**d**) and (**h**) Renilla luciferase activity was used for normalization and data is represented as fold activity with respect to control. Error bars in all the indicated sub-figures represent mean (+) s.d from three independent biological repeats. Indicated *P*-values were calculated using Student’s t-test and *P* < 0.0001 is represented as ****

To study whether the effect of p68 on RelA expression at the protein level is a consequence of its transcriptional regulation, we quantified its mRNA expression. In multiple CRC cell lines, p68 overexpression led to increase in mRNA expression of RelA (Fig. 2a). Conversely, upon p68 knockdown there was a significant reduction in RelA mRNA expression (Fig. 2b). Cyclin D1 (CCND1) served as a positive control for both [4]. These results indicate p68’s role in regulating RelA at the transcript level.

We next speculated that RelA promoter activity might be regulated by p68, as p68 is a co-activator of several oncogenic transcription factors that might bind on the RelA promoter. We studied the promoter region of RelA 2000 bp upstream to the transcriptional start site and found that this region contains seven putative β-catenin/TCF-LEF binding elements (TBEs). Wnt/β-catenin signaling is highly implicated in colon cancer progression and p68 is reported to interact with β-catenin and thereby, regulate the expression of its target genes [4, 5]. Hence, we hypothesized that p68 might regulate RelA gene expression via TBEs. To explore whether p68 positively modulated RelA gene expression by directly upregulating its promoter activity, we cloned 1746 bp fragment of the RelA promoter (231 bp upstream to the transcription start site). Our luciferase assay results demonstrated that p68 overexpression, significantly upregulated RelA promoter activity in HCT 116 and SW-480 cells (Fig. 2c). Conversely, the knockdown of p68 using multiple shRNAs and siRNAs showed prominent decrease of the RelA promoter activity in both HCT 116 and SW-480 cells (Fig. 2d and Additional file 1: Figure S1A). Hence, p68 elevates RelAexpression by transcriptional activation of its promoter.

Next, we conducted Chromatin Immunoprecipitation (ChIP) assay to gain deeper mechanistic insights into the modulation of RelA promoter activity mediated by p68 and β-catenin cooperation. Immunoprecipitations (IPs) were carried out using anti-p68, anti- β-catenin and anti-TCF4 followed by amplification of different TBEs sites on the RelA promoter region, as shown in the schematic diagram (Fig. 2e). The ChIP data in HCT 116 and SW-480 cells demonstrated that p68, β-catenin and TCF4 bind to the promoter region of RelA in vivo (Fig. 2f and Additional file 1: Figure S1B). Amplification of Cyclin D1 promoter region spanning TBEs sites served as a positive control. Upon p68 knockdown, the occupancy of β-catenin is sharply reduced (TBE site1–3) (Fig. 2g). This further highlights that p68 is indeed essential for the occupancy of β-catenin on the RelA promoter, for regulating its expression.

The RelA promoter possesses seven TBEs namely: TBE1 (AACAAAG; − 252 to − 259), TBE2 (CTTTGCT; − 329 to − 336), TBE3 (CGCAAAG; − 568 to − 575), TBE4 (CTTTGGG; − 973 to − 980), TBE5 (ACCAAAG; − 1268 to − 1275), TBE6 (CTTTGTG; − 1722 to − 1729) and TBE7 (CTTTGGG; − 1886 to − 1893). The positions are relative to the transcription start site. We generated RelA promoter constructs containing deletions (5 nucleotides deleted by SDM) of the seven TBEs, as shown in the schematic diagram (Additional file 1: Figure S1C). Results of the luciferase assays showed that deletion of the TBEs markedly reduced the RelA promoter activity in both HCT 116 and SW-480 cells. Deletion of TBE4 (TBEΔ4) and TBE7 (TBEΔ7) has the most prominent effect in HCT 116 cells. In SW-480 cells, increase of RelA promoter activity in the presence of p68 was almost abrogated in case of TBEΔ4, TBEΔ5, TBEΔ6 and TBEΔ7. Thus, p68 mainly exerts its function through β-catenin/TCF4 complex at TBE4 and TBE7 and all these sites are crucial for β-catenin-dependent regulation of the RelA promoter activity (Fig. 2h). Hence, we can conclude that p68 acts in association with β-catenin in mediating the transcriptional activation of the RelA promoter.

### β-Catenin regulates RelA gene expression in colon cancer

**Fig. 3:**
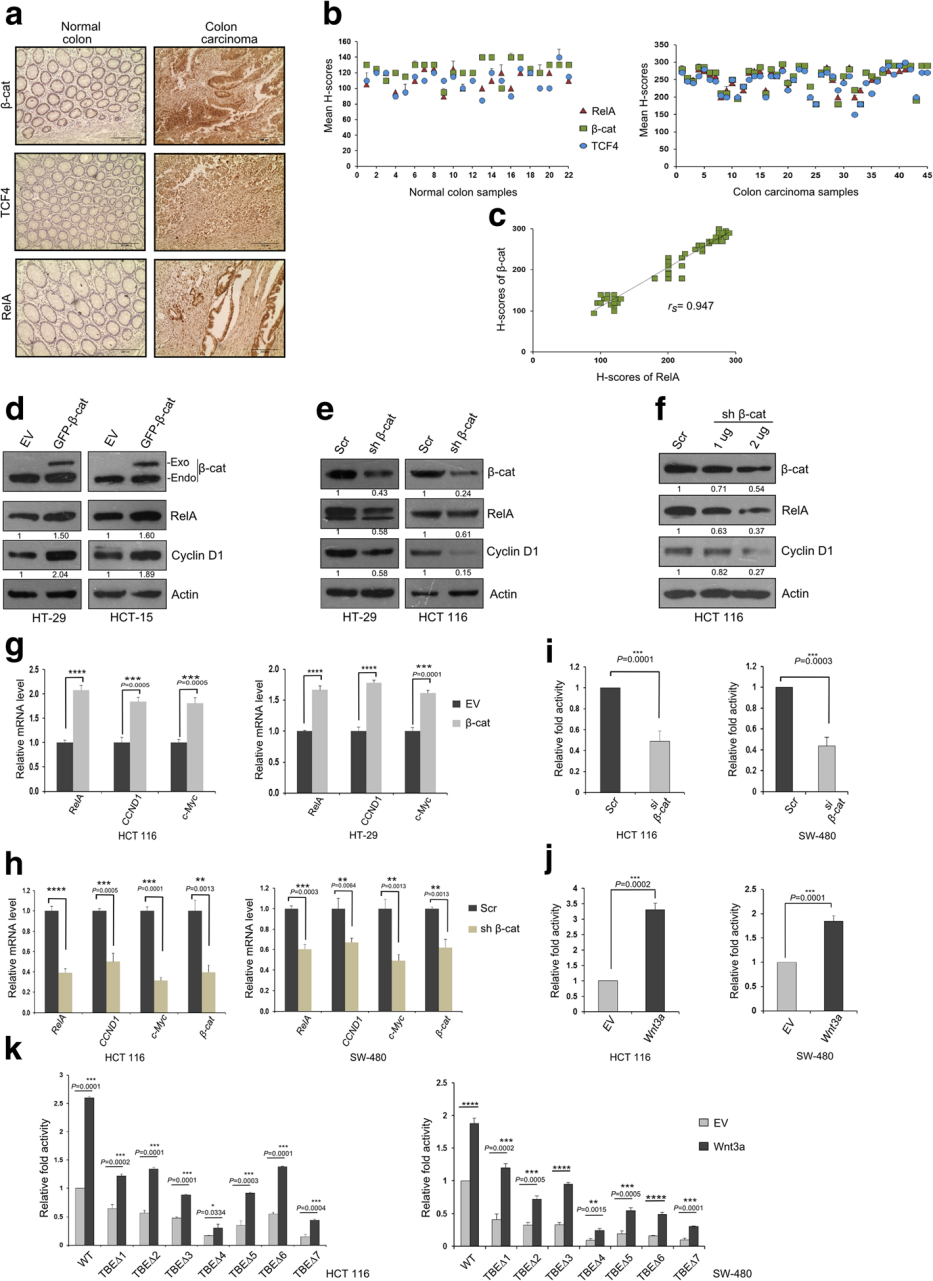
β-catenin regulates RelA gene expression in colon cancer cells**. a** Representative images depicting immunohistochemical (IHC) staining of β-catenin, TCF4 and RelA conducted in tissue sections derived from human normal colon and colon carcinoma samples. Images were captured at 200X magnification. **b** Scatter plots representing the mean H-scores of β-catenin, TCF4 and RelA in normal (*n* = 22) and colon carcinoma tissue (*n* = 45) samples, respectively. **c** Determination of Spearman’s rank correlation coefficient (rs) between the mean H-scores of β-catenin and RelA, analyzed from both normal and colon carcinoma tissues in combination. **d** Multiple colon cancer cell lines, grown in 60 mm cell culture dishes were transfected with 4 μg of pGZ-β-catenin or EV. Cell lysates were prepared, 36 h post transfection and probed for the indicated proteins by IB. ‘Exo’ and ‘Endo’ depicts exogenous and endogenous β-catenin, respectively. **e** Colon cancer cell lines, grown in 60 mm cell culture dishes were transfected with 4 μg of scr or β-catenin shRNA (represented as sh β-cat) plasmids. **f** HCT 116 cells were seeded in 60 mm plates and were transfected with scr or sh β-cat plasmids in a dose-dependent manner as indicated. For both (**e**) and (**f**) cell lysates were prepared, 48 h post transfection and probed for the indicated proteins by IB. Densitometry values below the blots in panels (**d**), (**e**) and (**f**) are relative to the loading control (Actin). **g** Colon cancer cell lines as indicated were seeded in 35 mm plates and transfected with 2 μg of pGZ- β-catenin or EV. Total RNA was extracted 36 h post transfection, followed by analysis of RelA mRNA by qRT–PCR. **h** Colon cancer cell lines as indicated were seeded in 35 mm plates and transfected with 2 μg of scr or sh β-cat. Total RNA was extracted, 48 h post transfection followed by analysis of RelA mRNA by qRT–PCR. For both (**g**) and (**h**) Cyclin D1 and c-Myc mRNA expression served as positive controls for β-catenin transfection. 18S rRNA was used for normalization. **i** HCT 116 (left) and SW-480 (right) cells were seeded in 35 mm plates and transfected with β-catenin siRNA (si β-cat,1 μl) or scr siRNA (control, 1 μl) along with pGL3-RelA-prom (1 μg) and Renilla luciferase plasmid (50 ng). The luciferase activity was measured, 48 h post transfection. **j** HCT 116 (left) and SW-480 (right) cells were seeded in 35 mm plates and co-transfected with either EV or pcDNA3.1-myc-his-Wnt3a (1 μg) along with pGL3-RelA-prom (1 μg) and Renilla luciferase construct (50 ng). **k** HCT 116 (left panel) and SW-480 (right panel) cells seeded in 35 mm plates were transfected with either pcDNA3.1-myc-his-Wnt3a or pcDNA3.1-myc-his (EV,1 μg) along with pGL3-WT-RelA-prom or its deletion constructs (1 μg) and Renilla luciferase plasmid (50 ng). For both (**j**) and (**k**) the luciferase activity was measured, 36 h post transfection. For (**i**), (**j**) and (**k**) cells co-transfected with (1 μg) of EV/control, pGL3-WT-RelA-prom and Renilla luciferase plasmid (50 ng) served as control. Renilla luciferase activity was used for normalization and data is represented as fold activity with respect to control cells. Error bars in all the indicated sub-figures represent mean (+) s.d from three independent biological repeats. Indicated P-values were calculated using Student’s t-test and *P* < 0.0001 is represented as ****

Canonical Wnt-β-catenin driven carcinogenesis is characterized by increased accumulation of β-catenin and TCF/LEF complexes in the nucleus leading to inappropriate activation of Wnt target genes; thus making β-catenin an enticing molecular target for CRC therapy [5]. From our previous results, we have established that p68 cooperates with β-catenin in regulation of RelA expression. We now wanted to study whether β-catenin has any direct role in regulating RelA. Hence, to investigate the possible connection of β-catenin with RelA, we conducted immunohistochemical (IHC) analysis in normal and colon carcinoma tissue samples procured from Indian patients. We also checked the expression status of TCF4, since TCF4 is the transcription factor to which β-catenin binds, on the promoter region of its target genes. The expression of β-catenin, TCF4 and RelA exhibited marked elevation in CRC samples compared to normal (Fig. 3a). The difference in staining intensities (measured by H-score) of β-catenin and TCF4 between normal and carcinoma samples (represented by Notched Box Plot) were extremely statistically significant, as predicted by the Mann–Whitney U-test analysis (Additional file 2: Figure S2A). The difference between the average H-scores of β-catenin and TCF4 in colon carcinoma samples was highly statistically significant as to that of the normal samples (Additional file 2: Figure S2B). Staining intensities of β-catenin, TCF4 and RelA (measured by H-score) followed similar trends in both normal and carcinoma samples (Fig. 3b). H-scores of β-catenin and RelA was found to bear strong positive correlation (rs = 0.947), when statistically analyzed by Spearman’s rank correlation test (Fig. 3c). These results indicate strong possible interconnection between β-catenin/TCF4 and RelA expression.

To investigate whether β-catenin regulates RelA gene expression in CRC cell lines, β-catenin was overexpressed in HT-29 and HCT-15. In both the cases, β-catenin enhanced RelA protein expression. Cyclin D1, a target gene of β-catenin [25] served as positive control for β-catenin overexpression, which also displayed similar trends of expression like RelA (Fig. 3d). Next, in a converse approach, we used short hairpin RNA (shRNA) against β-catenin to deplete its endogenous level. Significant reduction in RelA expression was observed upon knocking down β-catenin. Cyclin D1 followed a corresponding trend (Fig. 3e). To further delve into the possible effect of β-catenin on RelA, we decreased the endogenous level of β-catenin in a dose-dependent manner in HCT 116 cells using shRNA against it. The results demonstrated dose-dependent reduction in the expression of RelA, and also that of Cyclin D1 following a similar course (Fig. 3f). To study whether the effect of β-catenin on RelA at the protein level is a consequence of its transcriptional regulation, we quantified its mRNA expression. In multiple CRC cell lines, β-catenin overexpression led to increase in mRNA expression level of RelA (Fig. 3g and Additional file 3: Figure S3A). Conversely, upon β-catenin knockdown there was a significant reduction in RelA mRNA expression (Fig. 3h and Additional file 3: Figure S3B). Cyclin D1 and c-Myc (also a target gene of β-catenin) served as a positive control for both [26].

The requirement of p68 and β-catenin amalgamation in driving RelA gene expression is further supported by negation of β-catenin and p68 mediated increase in the mRNA levels of RelA in p68 or β-catenin depleted condition respectively (Additional file 3: Figure S3C and D). We next speculated that RelA promoter activity might be affected by β-catenin, as we have already established the occupancy of β-catenin on the promoter of RelA by ChIP assay. Our luciferase assay results demonstrated that upon knockdown of β-catenin endogenous level using either shRNA or siRNAs, a prominent decrease in RelA promoter activity was observed (Fig. 3i and Additional file 3: Figure S3E). Conversely, overexpression of β-catenin upregulated RelA promoter activity in both HCT 116 and SW-480 cells (Additional file 3: Figure S3F). Wnt3a, an important member of the Wnt family is a conserved, secreted signaling glycoprotein that influences cell behavior by stabilization and nuclear translocation of β-catenin. Substantial studies report Wnt3a expression to be significantly correlated with colorectal carcinoma’s clinical staging, metastasis and recurrence [27]. In this report, we observed Wnt3a overexpression led to significant upregulation of RelA promoter activity in both HCT 116 and SW-480 cells (Fig. 3j). Thus, based on the above results we could conclude that β-catenin elevates RelA expression by transcriptional activation of its promoter. Next, on Wnt3a and β-catenin overexpressed condition, luciferase assay was conducted using the seven mutant RelA promoters (one TBE site deleted in each mutant). Results of the luciferase assay were similar to that obtained on p68 overexpressed condition, i.e. marked reduction in RelA promoter activity, in both HCT 116 and SW-480 cells. Deletion of TBE4 (TBEΔ4) and TBE7 (TBEΔ7) showed the most prominent effect. Thus all the TBE sites are crucial for p68-β-catenin-dependent regulation of the RelA promoter activity especially sites TBE4 and TBE7 (Fig. 3k and Additional file 3: Figure S3G). Altogether, these results reinforce our hypothesis of p68 coactivating β-catenin/TCF4 complex in mediating the transcriptional activation of the RelA promoter.

### p68 and β-catenin synergism potentiates NF-κB signaling axis

**Fig. 4:**
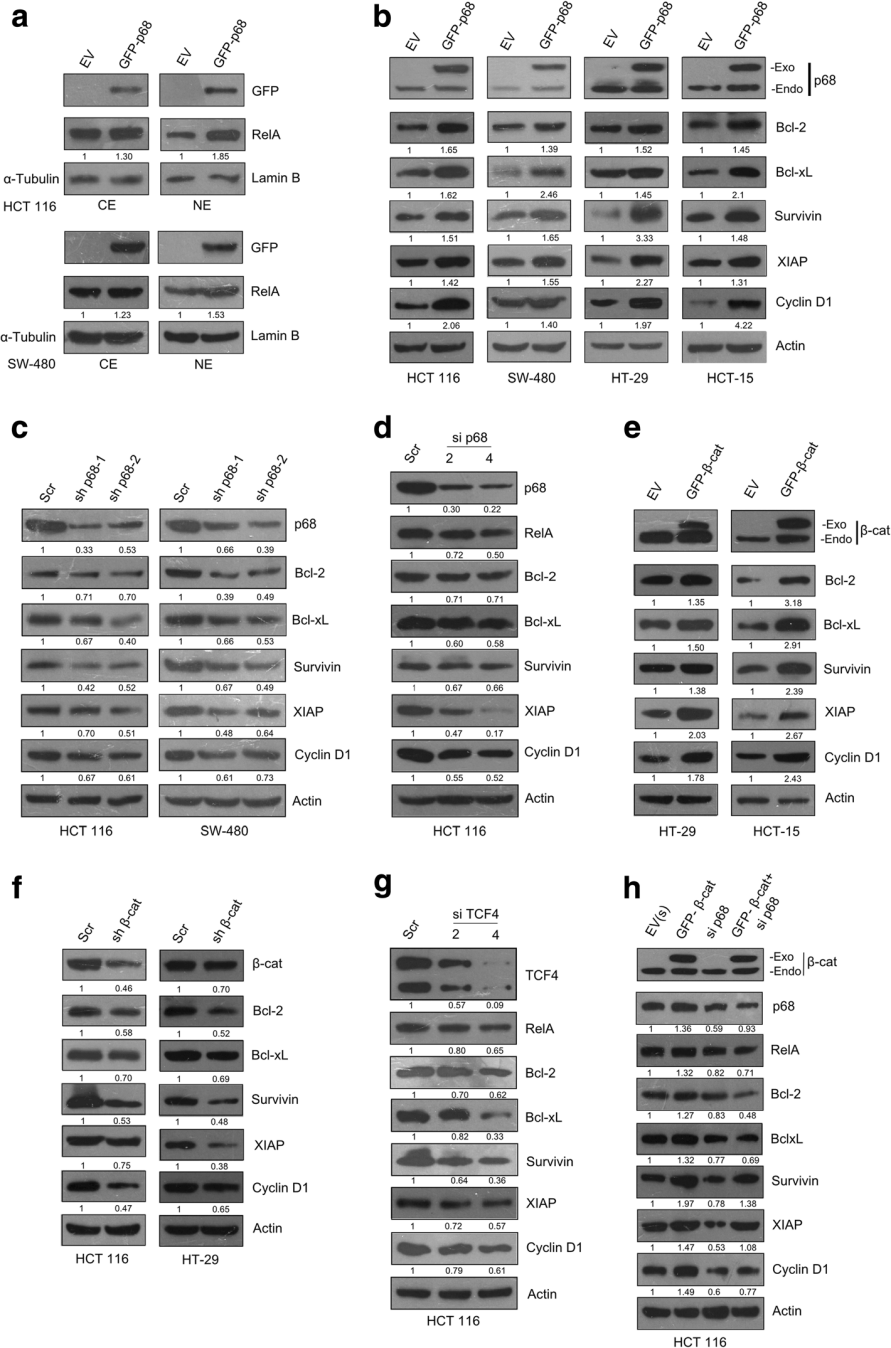
p68 and β-catenin synergism potentiates NF-κB signaling axis. **a** HCT 116 (upper panel) and SW-480 (lower panel) cells were seeded in 100 mm plates and transfected with 8 μg of EV or pGZ-p68. Cytoplasmic and nuclear extracts were prepared, 36 h post transfection; and probed for the indicated proteins by IB. Densitometry values relative to loading controls (α-tubulin for cytoplasmic and Lamin B for nuclear extract, respectively) are given below the blots. **b** Multiple colon cancer cell lines, grown in 60 mm cell culture dishes were transfected with 4 μg of pGZ-p68 or EV. ‘Exo’ and ‘Endo’ depicts exogenous and endogenous p68, respectively. **c** Colon cancer cell lines, as indicated were seeded in 60 mm cell culture dishes and transfected with 4 μg of scr or p68 shRNA1 and p68 shRNA2 constructs. **d** HCT 116 cells were seeded in 60 mm plates and were transfected with either scr or p68 siRNA in a dose-dependent manner as indicated. **e** Colon cancer cell lines, grown in 60 mm cell culture dishes were transfected with 4 μg of either pGZ-β-catenin or EV. ‘Exo’ and ‘Endo’ depicts exogenous and endogenous β-catenin, respectively. **f** Colon cancer cell lines, grown in 60 mm plates were transfected with 4 μg of either scr or β-catenin shRNA construct. **g** HCT 116 cells were seeded in 60 mm plates and were transfected with either scr or TCF4 siRNA in a dose-dependent manner as indicated. **h** HCT 116 cells were seeded in 60 mm plates and were transfected with 2 μg of either scr or p68 siRNA, in combination with 2 μg of pGZ- β-catenin or EV. Cell lysates were prepared from 36 h (**b** & **e**) and 48 h (**c**, **d**, **f**, **g** & **h**) post transfected cells and probed for the indicated proteins by IB as depicted in different sub-figures. Densitometry values below the blots in panels (**b-h**) are relative to the loading control (Actin). In sub-figures (**d** & **g**) - 2 and 4 μl siRNAs were used from 10 μM stock

Here, we pursued to investigate further, the outcome of p68-mediated RelA upregulation. In unstimulated cells, NF-κB complexes are ordinarily sequestered in the cytoplasm with inhibitory proteins IκBs. Upon stimulation, degradation of IκB, allows nuclear translocation of NF-κB complexes to promote the transcription of genes linked to a variety of physiological processes and multiple imperative aspects of oncogenesis [6]. We wanted to study whether p68 mediated upregulation of RelA has any effect on NF-κB signaling axis. We checked the nucleo-cytoplasmic distribution of RelA, in p68 overexpressed HCT 116 and SW-480 cells. The results clearly indicated an increase in the nuclear localization of RelA, implicating that this might have a role in upregulation and activation of NF-κB signaling pathway (Fig. 4a). From the above result, we postulated that p68 might have possible impact on NF-κB signaling axis as a result of RelA regulation. NF-κB as a transcription factor contributes to the progression of CRC by regulating the expression of diverse set of genes which act as a pivotal crossroad between cell proliferation and cell death; the two facets of the oncogenesis-governing equation. Bcl-2 [8], Bcl-xl [9], Survivin [11] and XIAP [10], regulated by NF-κB, act as prototype molecule at this crossroad and holds promise as target genes in CRC therapy [28, 29]. Overexpression of these genes is correlated with majority of the salient features of tumor development and progression [12, 13, 30]. To investigate further whether p68 affects the expression of NF-κB target genes, we checked the expression of genes responsible for enhanced survival (Survivin) and evasion of apoptosis (Bcl-2, Bcl-xL and XIAP). In multiple CRC cell lines upon p68 overexpression; all the selected NF-κB target genes displayed enhanced protein expression (Fig. 4b). In a converse approach, on depleting the endogenous level of p68 using shRNA against it, protein expression of all the selected NF-κB target genes markedly reduced in HCT 116 and SW-480 cell lines (Fig. 4c). Next, we depleted the endogenous level of p68 using siRNAs against it, in a dose dependent manner in HCT 116 cells. As anticipated the protein expression of RelA as well as, all the selected NF-κB target genes reduced in a dose dependent fashion (Fig. 4d). As we have already established the involvement of β-catenin in RelA gene regulation we wanted to investigate whether β-catenin also plays a role in activating the NF-κB signaling pathway, by regulating the expression of NF-κB target genes. Upon overexpression of β-catenin in HT-29 and HCT-15 cell lines, all the selected NF-κB target genes displayed enhanced protein expression (Fig. 4e). Depletion of the endogenous level of β-catenin using shRNA against it, led to reduction in the protein expression of the selected NF-κB target genes, in both HCT 116 and HT-29 cells (Fig. 4f). Since TCF4 is the transcription factor to which β-catenin binds, the ultimate downstream effector of Wnt/β-catenin signaling, we next checked the status of the selected NF-κB target genes upon depleting the endogenous level of TCF4 by using siRNAs against it in a dose dependent manner in HCT 116 cell line, as anticipated the expression of RelA along with the selected genes decreased in a dose dependent fashion (Fig. 4g). From the above results we could conclude that ‘p68- β-catenin/TCF4 complex’ plays an active role in regulating NF-κB signaling axis.

To gain deeper insights into p68 and β-catenin mediated regulation of NF-κB target genes; we pursued to explore further the interplay of p68 with β-catenin in regulating RelA-dependent gene expression. We observed that β-catenin mediated increase in the protein levels of RelA and the selected NF-κB target genes were abrogated in p68-depleted condition (Fig. 4h). This highlights that p68 and β-catenin collaboration is required for RelA expression, followed by NF-κB signal activation and expression of signature NF-κB dependent genes. In all the above experiments, Cyclin D1 served as a positive control for both p68 and β-catenin/TCF4 signaling. Thus the above results corroborate that; p68 along with β-catenin/TCF4 exerts a strong influence on NF-κB signaling pathway.

### p68 and RelA maintain positive correlation with NF-κB target genes in colon carcinoma samples

**Fig. 5:**
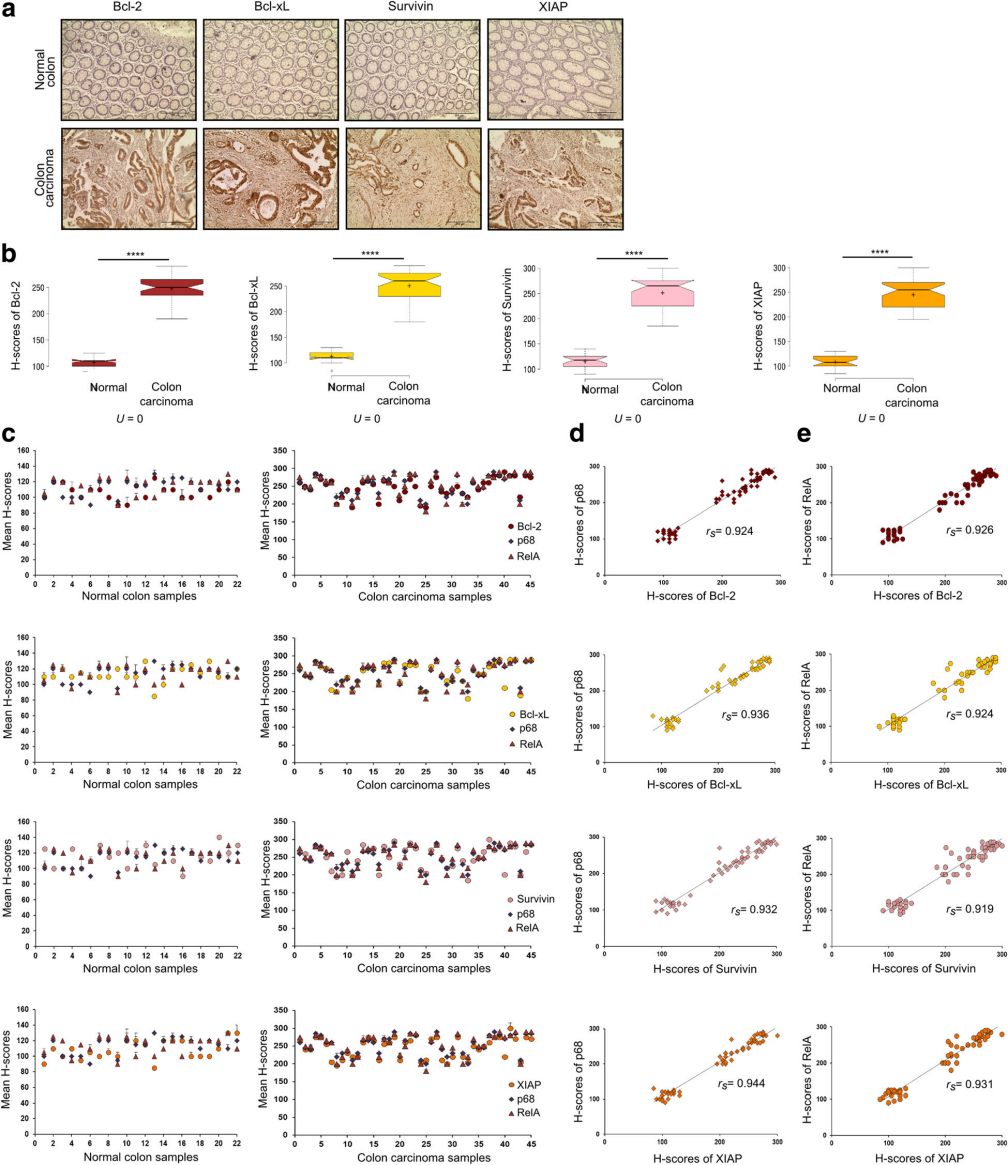
Expression of NF-κB target genes, p68 and RelA is positively correlated in human colon cancer tissues. **a** Representative images depicting IHC staining of Bcl-2, Bcl-xL, Survivin and XIAP conducted in tissue sections derived from human normal colon and colon carcinoma samples. Images were captured at 200X magnification. **b** Notched box plots showing the distribution of H-scores of Bcl-2, Bcl-xL, Survivin and XIAP in normal (*n* = 22) and colon carcinoma (*n* = 45) samples; Mann–Whitney U-values were calculated from H-scores. **c** Scatter plots representing the mean H-scores of p68, RelA and NF-κB target genes (Bcl-2, Bcl-xL, Survivin and XIAP) in normal and colon carcinoma tissue samples, respectively. **d** Spearman’s rank correlation coefficient (rs) between the mean H-scores of p68 and (**e**) RelA with each of the NF-κB target genes as indicated in the sub-figures was determined from both normal and colon carcinoma tissues in combination

After having established the involvement of p68 and β-catenin synergism in regulating NF-κB signaling pathway, we were inclined to investigate the clinical relevance of this signaling cascade. To inspect the expression status of the NF-κB target genes in human colon carcinoma tissue samples, we performed IHC analysis of Bcl-2, Bcl-xL, Survivin and XIAP in the same set of normal and colon carcinoma tissue samples, used in Figs. 1a and 3a. The abundance of Bcl-2, Bcl-xL, Survivin and XIAP were enhanced in colon carcinoma samples compared to normal (Fig. 5a). The difference in staining intensities as measured by H-score, of these proteins between normal and carcinoma samples were also statistically significant, as predicted by the Mann–Whitney U-test analysis, signifying the activation of these genes in the genesis stage of colorectal carcinoma (Fig. 5b). H-scores of p68 and RelA followed similar trends with H-scores of Bcl-2, Bcl-xL, Survivin and XIAP in both normal and carcinoma samples (Fig. 5c). H-scores of p68 were found to bear strong positive correlation with H-scores of Bcl-2 (rs = 0.924), Bcl-xL (rs = 0.936), Survivin (rs = 0.932) and XIAP (rs = 0.944) (Fig. 5d); likewise H-scores of RelA were found to bear strong positive correlation with H-scores of Bcl-2 (rs = 0.926), Bcl-xL (rs = 0.924), Survivin (rs = 0.919) and XIAP (rs = 0.931) when statistically analyzed by Spearman’s rank correlation test (Fig. 5e). These results indicate highly conceivable interconnection between p68 and NF-κB target genes-Bcl-2, Bcl-xL, Survivin and XIAP.

To widen our study horizon in terms of clinical relevance of this signaling cascade we further investigated the relationship between the expression status of β-catenin with p68 and the NF-κB target genes. Interestingly, H-scores of β-catenin were also found to follow similar trends with H-scores of p68 and NF-κB target genes-Bcl-2, Bcl-xL, Survivin and XIAP in both normal and carcinoma samples (Additional file 4: Figure S4A). H-scores of β-catenin bearing strong positive correlation with H-scores of p68 (rs = 0.958) and downstream NF-κB target genes-Bcl-2 (rs = 0.923), Bcl-xL (rs = 0.931), Survivin (rs = 0.962) and XIAP (rs = 0.937) (Additional file 4: Figure S4B). These results augment our in vitro findings of enhancement of NF-κB signaling axis by p68 and β-catenin collaboration.

### Primary tumors of murine colorectal allograft model stably expressing p68 show augmented expression of RelA and NF-κB target genes

**Fig. 6:**
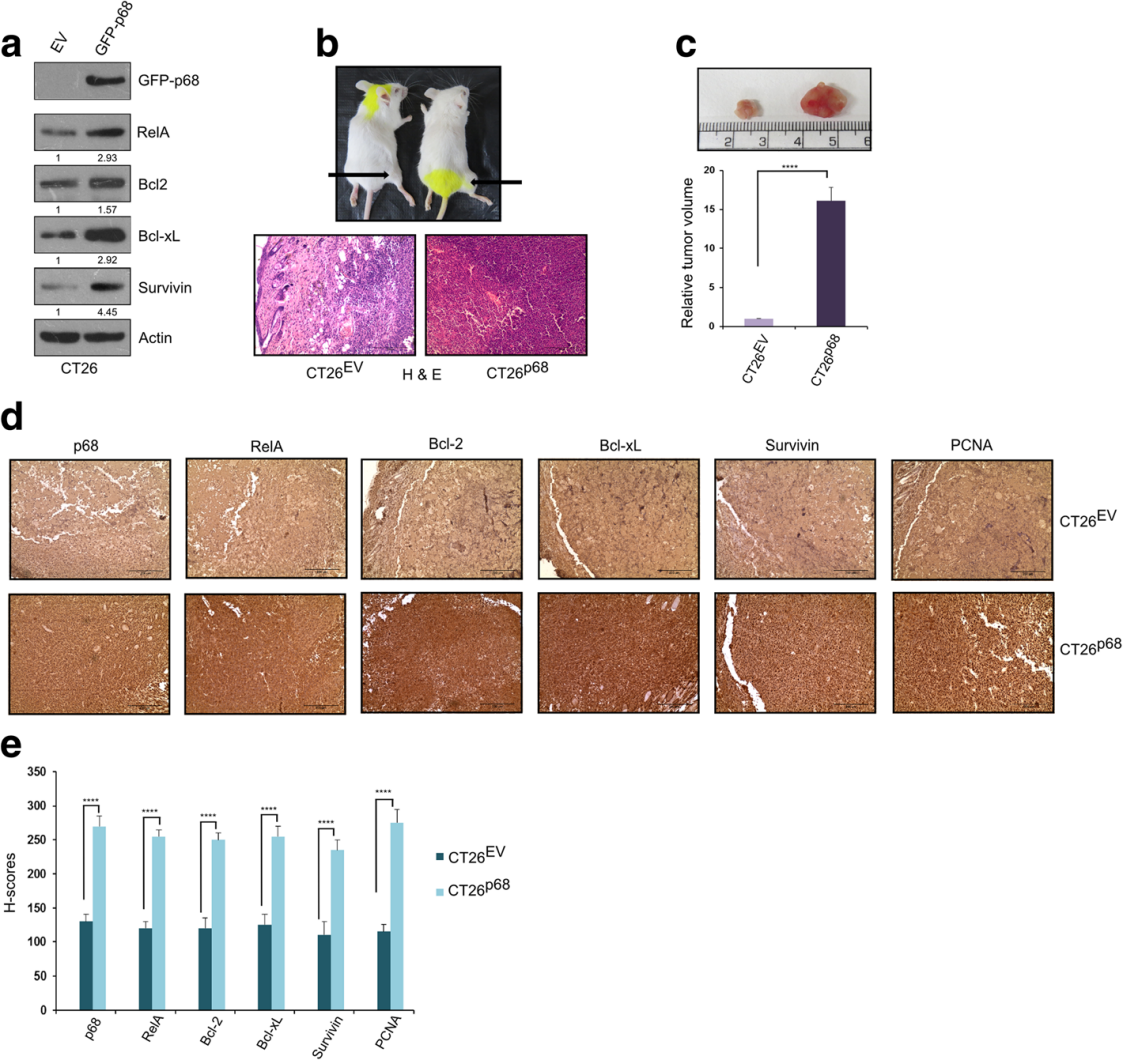
p68 overexpression in mice colorectal allograft model leads to enhanced primary tumor growth. **a** Mice syngeneic colon cancer cells-CT26 was transfected with either 4 μg of pEGFP-p68 or EV. G418 (500 μg/ml) was used for selection and maintenance of stable cells (CT26^EV^ and CT26^p68^). Cell lysates were prepared, and probed for the indicated proteins by IB. Densitometry values relative to loading control (Actin) are given below the blots. **b** Representative images of the mice bearing the primary tumor generated after 30 days of inoculation of stable CT26^EV^ and CT26^p68^ cells into the flank region of BALB/c (top panel). Arrows indicate the tumors. Corresponding H&E-stained images of the respective tumor sections captured at 200X magnification (bottom panel). **c** Representative images of the generated primary tumors (top panel). Data in the bottom panel represents the tumor volume generated from stable CT26^p68^ cells relative to the CT26^EV^ cells. **d** Representative images depicting IHC staining of p68, RelA, Bcl-2, Bcl-xL Survivin and PCNA conducted in mice tumor-derived tissue sections. Images were captured at 200X magnification. **e** Comparison of the average H-scores of the indicated proteins. Error bars in (**c** [bottom panel] & **e**) represent mean (+) s.d from independent biological repeats (*n* = 6). *P* < 0.0001 is represented as **** (Student’s t-test)

We extended our investigation in mice colorectal tumor to assess the physiological relevance of our in vitro findings. Primary tumors were generated using mice syngeneic adenocarcinoma cell line CT26, stably expressing either enhanced green fluorescent protein (EGFP)-p68 (CT26^p68^) or the empty vector encoded EGFP (CT26^EV^). The murine CT26 is a grade IV carcinoma cell line developed in 1975 by exposing BALB/c mice to N-nitroso-N-methylurethane (NMU) and is extensively used as a model system in investigation of colorectal carcinomas. The expression of RelA and NF-κB target genes were significantly elevated in CT26^p68^ cells compared with CT26^EV^ (Fig. 6a). The primary tumor volume produced after 30 days from CT26^p68^ cells was markedly higher (approximately 15 times) with enhanced vasculature than that produced from CT26^EV^ cells (Fig. 6c). Hematoxylin and eosin (H&E) staining was conducted to examine the gross morphological features of the tissue sections derived from these tumors and it revealed, the tumor mass formed by CT26^p68^ to be large and more compact with higher cellular density in comparison to CT26^EV^ (Fig. 6b and c). IHC analysis showed heightened expression of p68, RelA and NF-κB target genes- Bcl-2, Bcl-xL and Survivin in the tissue sections derived from CT26^p68^ tumors. PCNA served as proliferation marker, also showed increased expression (Fig. 6d). Highly significant difference was exhibited in the H-scores of each of the proteins in CT26^p68^ tumor-derived tissues in comparison to CT26^EV^ (Fig. 6e). Taken together, these animal data suggest that p68 enhances primary tumor growth, leads to upregulation of RelA and subsequent activation of oncogenic NF-κB signaling axis and corroborates the correlation between p68 and expression of NF-κB target genes.

### p68 triggers expression of NF-κB target genes in lung metastatic nodules

**Fig. 7:**
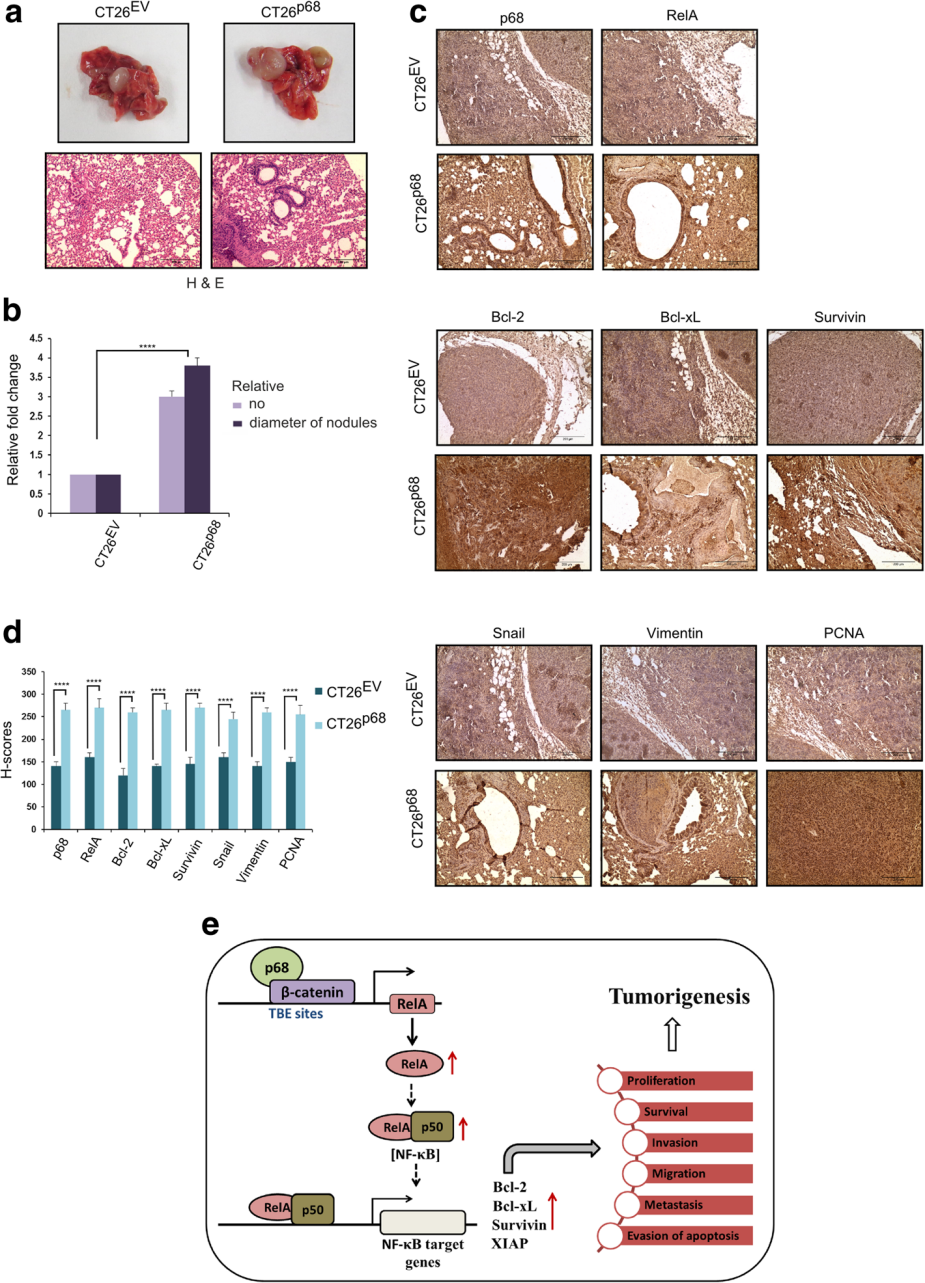
p68 overexpression augments lung metastasis. **a** Representative images of lungs with metastatic nodules developed after 30 days of inoculation of stable CT26^EV^and CT26^p68^ cells into the tail vein of BALB/c mice; *n* = 6 (top panel). Corresponding H&E-stained images of lung tissue sections containing metastatic nodules captured at 200X magnification (bottom panel). **b** Data represents the number and diameter of metastatic nodules in the lungs derived from CT26^p68^ cells relative to the CT26^EV^ cells. **c** Representative images depicting IHC staining of p68, RelA (top); Bcl-2, Bcl-xL, Survivin (middle) and Snail, Vimentin and PCNA (bottom) conducted in lung metastatic nodules containing tumor tissue sections. Images were captured at 200X magnification. **d** Comparison of the average H-scores of the indicated proteins. Error bars in (**b** & **d**) represent mean (+) s.d. from independent biological repeats (*n* = 6); *P* < 0.0001 is represented as **** (Student’s t-test). **e** Model: schematic representation, depicting p68 coactivation of β-catenin in upregulation of RelA transcriptional activity and the subsequent effect on NF-κB signaling driven tumorigenesis in colon cancer. The inceptive event is triggered by p68, it cooperates with β-catenin in occupying the TBE sites in the promoter of RelA and significantly upregulates RelA transcription. This escalates RelA import to the nucleus leading to enhanced NF-κB complex activation and potentiates its transcriptional activity, manifested by the upregulation of its target genes such as Bcl-2, Bcl-xL, Survivin and XIAP. Elevated expression of these proto-oncogenes paves the way for enhanced rate of cell proliferation, survival, invasion, migration, metastasis and evasion of apoptosis, thus cumulatively contributing to oncogenesis

To investigate the impact of p68 in development of lung metastasis, we injected stable CT26^p68^ and CT26^EV^ cells intravenously into the tail vein of BALB/c mice. After 30 days of inoculation, it was observed that the metastatic lung nodules developed in mice injected with stable CT26^p68^ were much higher in number (approximately three times) and elevated in size (approximately four times) and possessed compact tissue morphology with high cellular density as observed by the histological analysis (H&E staining) of the lung tissue sections in comparison to that formed by CT26^EV^ cells (Fig. 7a and b). IHC analysis showed elevated expression of p68, RelA, NF-κB target genes- Bcl-2, Bcl-xL and Survivin in the tissue sections of CT26^p68^-derived lung metastatic nodules. Proliferation marker PCNA also displayed heightened expression in the CT26^p68^-derived metastatic nodules. EMT is characterized with increase expression of EMT markers like Vimentin and Snail; we observed augmented expression of these proteins in the lung tumor tissue derived from the mice intravenously injected with CT26^p68^ in comparison to CT26^EV^ cells (Fig. 7c). Each of the examined proteins in CT26^p68^ tumor-derived tissues of lung metastatic nodules exhibited marked difference in H-score from CT26^EV^ tumor-derived tissues (Fig. 7d). Thus, p68-mediated upregulation of RelA and consequent activation of NF-κB signaling was followed by the elevated expression of NF-κB target genes henceforth accelerating the process of tumor formation and metastasis.

## Discussion

High prevalence of CRC demands in depth understanding of the molecular mechanism underlying its initiation and progression. A significant percentage of cancer especially CRC results from chronic inflammation, a key mediator of which are components of NF-κB signaling [7]. Multiple lines of evidence suggest constitutively activated NF-κB (RelA) expression in human CRC tissue [15, 31]. NF-kB plays a preponderant role in CRC development and progression, also reported to promote resistance to chemo- and radio- therapies, thus marking itself an interesting therapeutic target for treatment of cancer and demands further investigation [32]. A better understanding of the complex functional mechanism behind the regulation of RelA and NF-κB signaling cascade may provide opportunities for development of effective therapeutic strategy and open new avenues in the area of translational oncology. In this study, we revealed compelling evidences of p68 mediated RelA gene regulation and subsequent activation of NF-κB signaling axis in colon cancer.

A strong positive correlation was perceived between the abundance of p68 and RelA in both normal and colon carcinoma tissue samples. RelA protein and mRNA levels were enhanced by p68 in colon cancer cells. Analysis of the RelA promoter revealed presence of TBEs sites. Results of Luciferase assay demonstrated, p68-mediated transcriptional activation of the RelA promoter. Conversely, reduction of RelA promoter activity was observed on p68 knockdown condition. Our ChIP result established occupancy of p68 and β-catenin on the RelA promoter in vivo. Also interestingly, β-catenin’s binding on the RelA promoter was severely hampered in p68 knockdown condition thus corroborating the considerable dependency of β-catenin on p68 and the crucial role played by p68 in β-catenin mediated RelA gene regulation. Mutation of different TBE sites revealed that TBE4 and TBE7 played the most significant role in p68-β-catenin driven RelA gene regulation. The effect of mutation was more prominent in SW-480 cells signifying a stronger dependency of SW-480 cells on p68-β-catenin mediated regulation of RelA compared to HCT 116 cells.

The interplay between Wnt/β-catenin and NF-κB/RelA is complicated and is mediated by regulations and interactions at multiple cellular levels [18, 33, 34]. Also, simultaneous activation of both the signaling pathways, neither alone is required for enhanced breast cancer stem cell (CSC) phenotypes [35]. Understanding the molecular mechanism behind this crosstalk and its role in CRC progression is an interesting area of investigation. Our results demonstrated the occupancy of β-catenin on the promoter of RelA, hence we next explored the involvement of Wnt/β-catenin signaling in RelA gene expression. Strong positive correlation was observed between the abundance of β-catenin, TCF4 and RelA in both normal and colon carcinoma tissue samples. β-catenin regulated RelA at the promoter level, hence RelA’s protein and mRNA levels were enhanced by β-catenin. Wnt3a and β-catenin overexpression led to significant upregulation of RelA promoter activity which substantially diminished on mutation of TBE sites on the RelA promoter especially on mutation of TBE4 and TBE7. This result was endorsed by the effect observed on p68 over expressed condition; thus reinforcing our hypothesis of p68 being the coactivator of β-catenin in transcriptional activation of the RelA promoter.

To investigate the consequences of p68-β-catenin mediated RelA upregulation, we checked the nuclear distribution of RelA, which was found to be enhanced on p68 over expression. This implies activation of NF-κB signaling axis; however the mechanistic insight behind this crucial phenomenon requires further experimental dissections. NF-κB as a transcription factor is reported to govern all six hallmarks of cancer by regulating the expression of a diverse array of genes involved in cell survival, proliferation, angiogenesis, evasion from apoptosis, invasion and inflammation. On exploring the sequel of NF-κB activation, we found that p68 enhanced protein levels of NF-κB target genes- Bcl-2, Bcl-xL, Survivin and XIAP. Signal crosstalk between β-catenin and NF-κB represents a complex functional network and how it contributes to the regulated expression of their target genes is a matter of great concern and needs further comprehensive dissection. Interestingly, β-catenin mediated increase in the protein levels of RelA and NF-κB target genes were abolished in p68-depleted condition, highlighting p68 to be the predominant factor in β-catenin/TCF4 conciliated RelA expression and subsequent activation of NF-κB signaling. We found enhanced expression of these proteins in CRC samples and a strong positive correlation was observed between the abundance of p68, RelA, β-catenin and studied NF-κB target genes.

The current study uncovers p68 as an oncogenic signaling intermediate, connecting NF-κB and Wnt/β-catenin signaling pathways to regulate plethora of genes required for oncogenesis. This marks p68 as a crucial target for therapeutic interventions. Further efforts to decipher the dynamic role played by p68 will help to untangle the contribution of complex intricate cellular signaling mechanism and their cross-regulation in promoting tumorigenesis.

## Conclusions

In summary, our study illustrates a novel mechanism of p68 mediated deployment of β-catenin in RelA gene regulation eventually leading to activation and recruitment of NF-κB signaling axis in the cell survival pathway for promoting tumorigenesis. A schematic representation of the proposed mechanism is portrayed in Fig. 7e. We propose that p68 coactivates β-catenin in occupying the TBE sites on the RelA promoter and increases its transcription. Increased abundance of RelA protein eventually leads to activation of NF-κB signaling. These strings of events collectively result in heightened expression of signature NF-κB target genes that tip the balance between proliferation and apoptosis towards achieving the pinnacle in oncogenesis.

## Additional files

Additional file 1: **Figure S1.** p68 regulates RelA gene expression**. (A)** HCT 116 (left) and SW-480 (right) cells were seeded in 35 mm plates and transfected with p68 siRNA (1 μl) or scramble siRNA (scr, 1 μl) along with pGL3-RelA-prom (1 μg) and Renilla luciferase plasmid (50 ng). The luciferase activity was measured 48 h post transfection. Renilla luciferase activity was used for normalization and the data is represented as fold activity with respect to control (scr). Error bars represent mean (+) s.d. Indicated *P*-values were determined using Student’s t-test. Scramble and p68 siRNA were used at a final concentration of 30 nM. **(B)** SW-480 cells were grown in 100 mm cell culture dishes followed by Chromatin immunoprecipitation (ChIP) using the indicated antibodies. ChIP using antibody against RNA Polymerase II (Pol II) and IgG served as positive and negative controls, respectively. Amplification of Cyclin D1 promoter containing TCF-binding elements (TBE) served as positive control for both p68 and β-catenin. Actin promoter served as positive control for Pol II. DNA extract (10% without ChIP) was used as input. PCR amplification of the immunoprecipitated DNA was performed using primers designed from the RelA promoter region – TBE [1–3]-(349 bp), flanking TBE1, TBE2 and TBE3; TBE4-(296 bp), flanking TBE4; TBE5-(140 bp), flanking TBE5 and TBE [6–7]-(213 bp), flanking TBE6 & TBE7. **(C)** Schematic representation of the RelA promoter reporter constructs (WT and mutated). White boxes represent TBEs and black boxes represent mutated TBE (5 nucleotides deleted by SDM). The numbers indicate the position of each TBE with respect to transcription start site. (TIF 385 kb)

Additional file 2: **Figure S2.** β-catenin and TCF4 expression is elevated in CRC samples. **(A)** Notched box plots showing the distribution of H-scores of β-catenin and TCF4 in normal (*n* = 22) and colon carcinoma samples (*n* = 45); Mann–Whitney U-values were calculated from H-scores. **(B)** Comparison of the combined average H-scores of β-catenin and TCF4. Error bars represent the mean (+) s.d.; *P* < 0.0001 is represented as ****; calculated using Student’s t-test. (TIF 106 kb)

Additional file 3: **Figure S3.** Amalgamation between p68 and β-catenin regulates RelA expression. (**A**) SW-480 cells seeded in 35 mm plates were transfected with pGZ-β-catenin or EV (2 μg). (**B**) HCT-15 cells seeded in 35 mm plates were transfected with scr or β-catenin shRNA (2 μg). HCT 116 cells seeded in 35 mm plates were transfected with either (**C**) scr or p68 shRNA-1 (1 μg), in combination with pGZ-β-catenin or EV (1 μg) or (**D**) scr or β-catenin shRNA (1 μg), in combination with pGZ-p68 or EV (1 μg). Total RNA was extracted; 36 h post transfection for (**A**) and 48 h post transfection for (**B**), (**C**) and (**D**), followed by analysis of RelA mRNA by qRT–PCR. 18S rRNA was used for normalization in all the above experiments. HCT 116 (left) and SW-480 (right) cells seeded in 35 mm plates were transfected with (**E**) scr or β-catenin shRNA plasmid (1 μg) along with pGL3-RelA-prom (1 μg) and Renilla luciferase plasmid (50 ng). The luciferase activity was measured, 48 h post transfection. (**F**) EV or pGZ-β-catenin (1 μg) along with pGL3-RelA-prom (1 μg) and Renilla luciferase construct (50 ng). (**G**) pGZ-β-catenin or pGZ EV (1 μg) along with pGL3-WT-RelA-prom or its deletion constructs (1 μg) and Renilla luciferase plasmid (50 ng). For both (**F**) and (**G**) the luciferase activity was measured, 36 h post transfection. For (**E**), (**F**) and (**G**) cells co-transfected with (1 μg) of EV, pGL3-WT-RelA-prom and Renilla luciferase plasmid (50 ng). Renilla luciferase activity was used for normalization and data is represented as fold activity with respect to control cells. For all the sub-figures results are presented as mean (+) s.d. from three independent experiments. Indicated P-values were determined using Student’s t-test and *P* < 0.0001 is represented as ****. (TIF 407 kb)

Additional file 4: **Figure S4.** Abundance of β-catenin with p68 and NF-κB target genes maintain strong positive correlation in human colon cancer tissues. **(A)** Scatter plots representing the mean H-scores of β-catenin and p68, Bcl-2, Bcl-xL, Survivin and XIAP in normal (*n* = 22) and colon carcinoma tissue (*n* = 45) samples, respectively. **(B)** Spearman’s rank correlation coefficient (rs) between the mean H-scores of β-catenin and p68, Bcl-2, Bcl-xL, Survivin and XIAP was determined from both normal and colon carcinoma tissues in combination. (TIF 628 kb)

Additional file 5: **Table S1.** List of primers used in the study. (DOCX 19 kb)

## Data Availability

All data generated and analyzed during this study are included in this manuscript and its additional files.

## Abbreviations

Bcl-2: B-cell lymphoma 2
Bcl-xL: B-cell lymphoma-extra large
CRC: Colorectal cancer
DMEM: Dulbecco’s Modified Eagle’s Medium
EMT: Epithelial-to-mesenchymal transition
FBS: Fetal bovine serum
FFPE: Formalin-fixed and paraffin-embedded
GAPDH: Glyceraldehyde-3-phosphate Dehydrogenase
GFP: Green fluorescent protein
H&E: Hematoxylin and eosin
HRP: Horse radish peroxidase
IgG: Immunoglobulin G
IHC: Immunohistochemistry
NF-κB: Nuclear factor-κB
PCR: Polymerase Chain Reaction
XIAP: X-linked inhibitor of apoptosis
