# Supplementary figures and images for: RNA helicase p68 deploys β-catenin in regulating RelA/p65 gene expression: implications in colon cancer
Source: J Exp Clin Cancer Res. 2019 Jul 27;38:330. doi: 10.1186/s13046-019-1304-y (PMC6660689; doi:10.1186/s13046-019-1304-y)

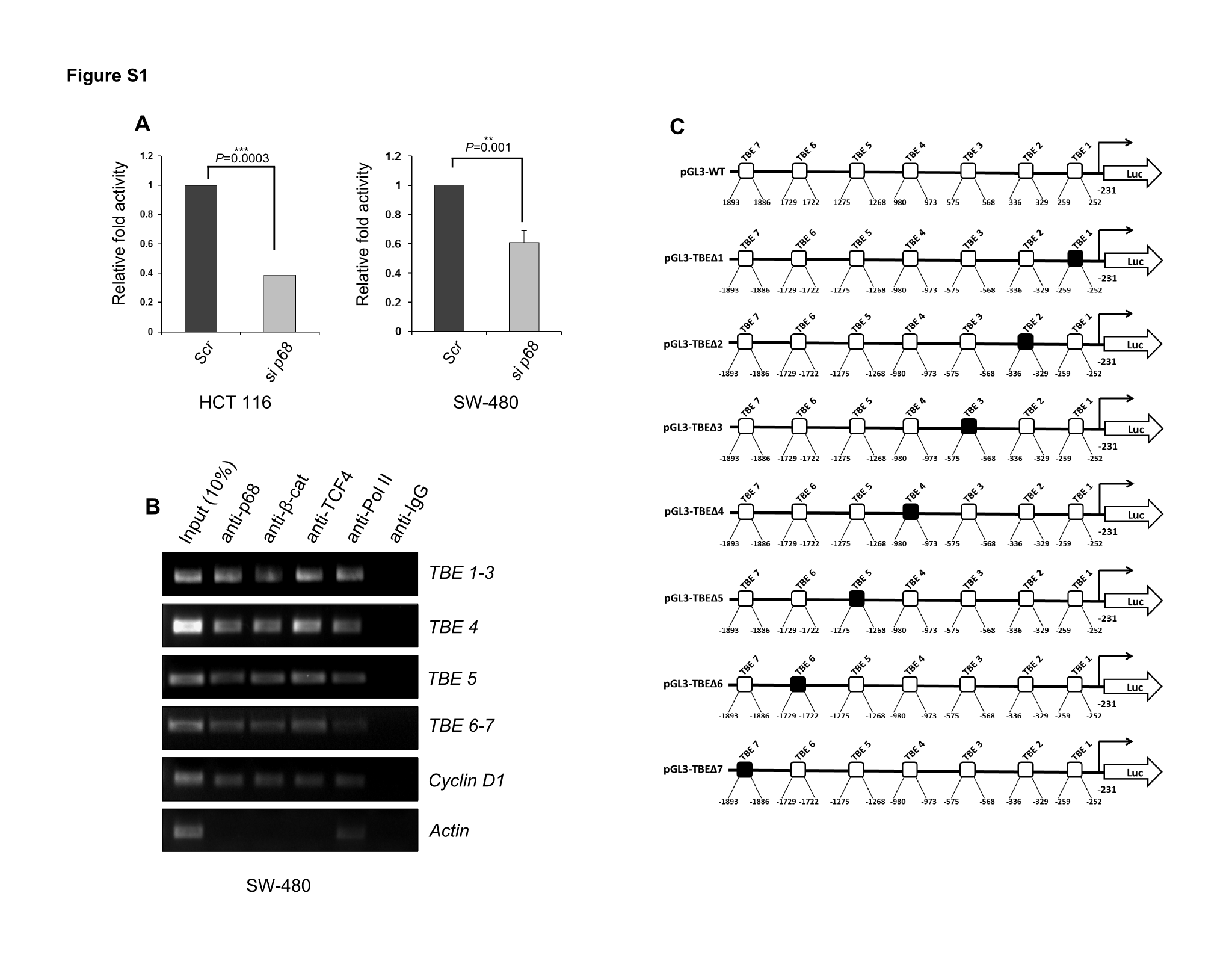

Supplement: Supplementary file 1 — Figure S1. p68 regulates RelA gene expression. (A) HCT 116 (left) and SW-480 (right) cells were seeded in 35 mm plates and transfected with p68 siRNA (1 μl) or scramble siRNA (scr, 1 μl) along with pGL3-RelA-prom (1 μg) and Renilla luciferase plasmid (50 ng). The luciferase activity was measured 48 h post transfection. Renilla luciferase activity was used for normalization and the data is represented as fold activity with respect to control (scr). Error bars represent mean (+) s.d. Indicated P-values were determined using Student’s t-test. Scramble and p68 siRNA were used at a final concentration of 30 nM. (B) SW-480 cells were grown in 100 mm cell culture dishes followed by Chromatin immunoprecipitation (ChIP) using the indicated antibodies. ChIP using antibody against RNA Polymerase II (Pol II) and IgG served as positive and negative controls, respectively. Amplification of Cyclin D1 promoter containing TCF-binding elements (TBE) served as positive control for both p68 and β-catenin. Actin promoter served as positive control for Pol II. DNA extract (10% without ChIP) was used as input. PCR amplification of the immunoprecipitated DNA was performed using primers designed from the RelA promoter region – TBE [1–3]-(349 bp), flanking TBE1, TBE2 and TBE3; TBE4-(296 bp), flanking TBE4; TBE5-(140 bp), flanking TBE5 and TBE [6–7]-(213 bp), flanking TBE6 & TBE7. (C) Schematic representation of the RelA promoter reporter constructs (WT and mutated). White boxes represent TBEs and black boxes represent mutated TBE (5 nucleotides deleted by SDM). The numbers indicate the position of each TBE with respect to transcription start site. (TIF 385 kb) [file 13046_2019_1304_MOESM1_ESM.tif]

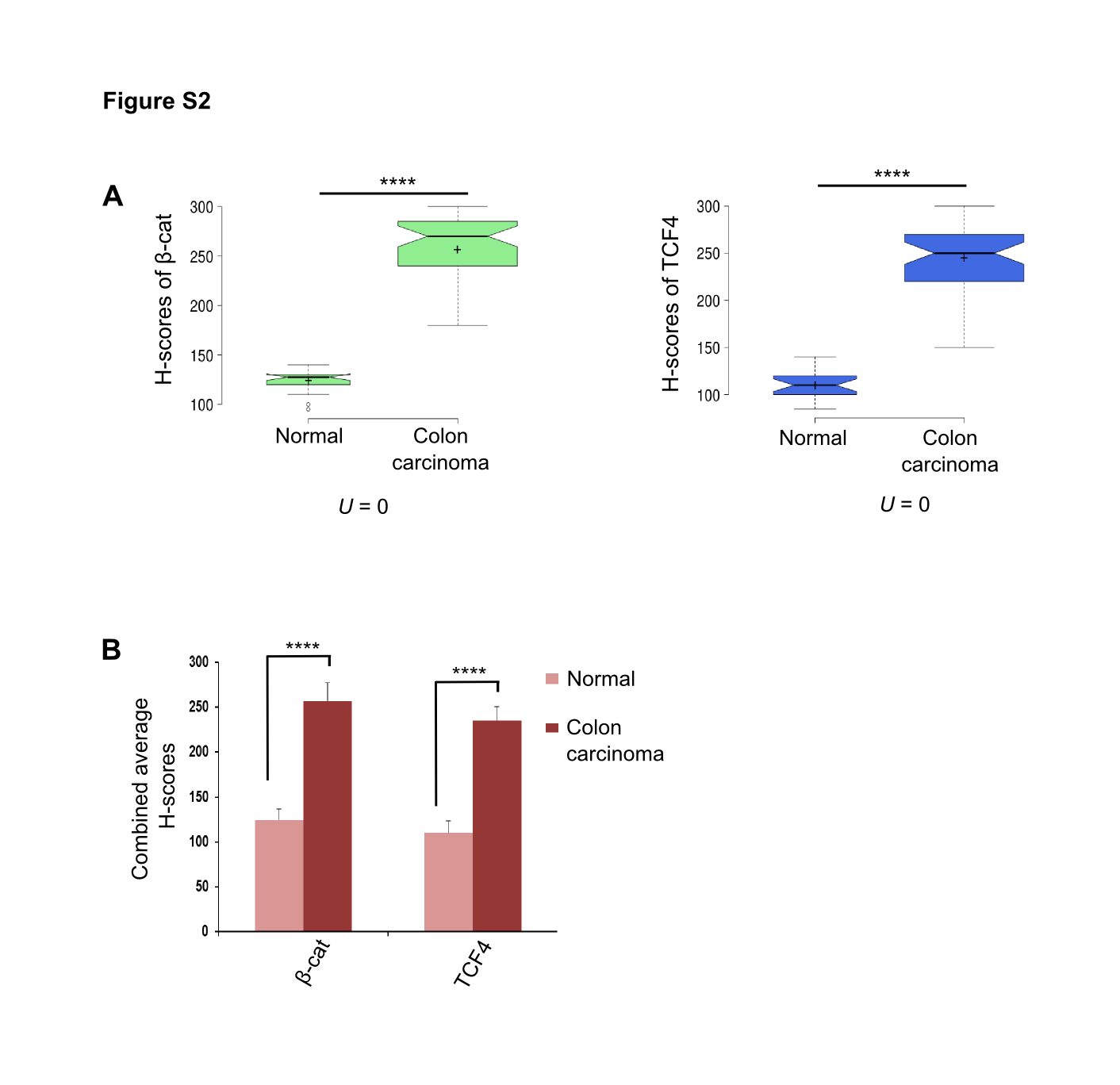

Supplement: Supplementary file 2 — Figure S2. β-catenin and TCF4 expression is elevated in CRC samples. (A) Notched box plots showing the distribution of H-scores of β-catenin and TCF4 in normal (n = 22) and colon carcinoma samples (n = 45); Mann–Whitney U-values were calculated from H-scores. (B) Comparison of the combined average H-scores of β-catenin and TCF4. Error bars represent the mean (+) s.d.; P < 0.0001 is represented as ****; calculated using Student’s t-test. (TIF 106 kb) [file 13046_2019_1304_MOESM2_ESM.tif]

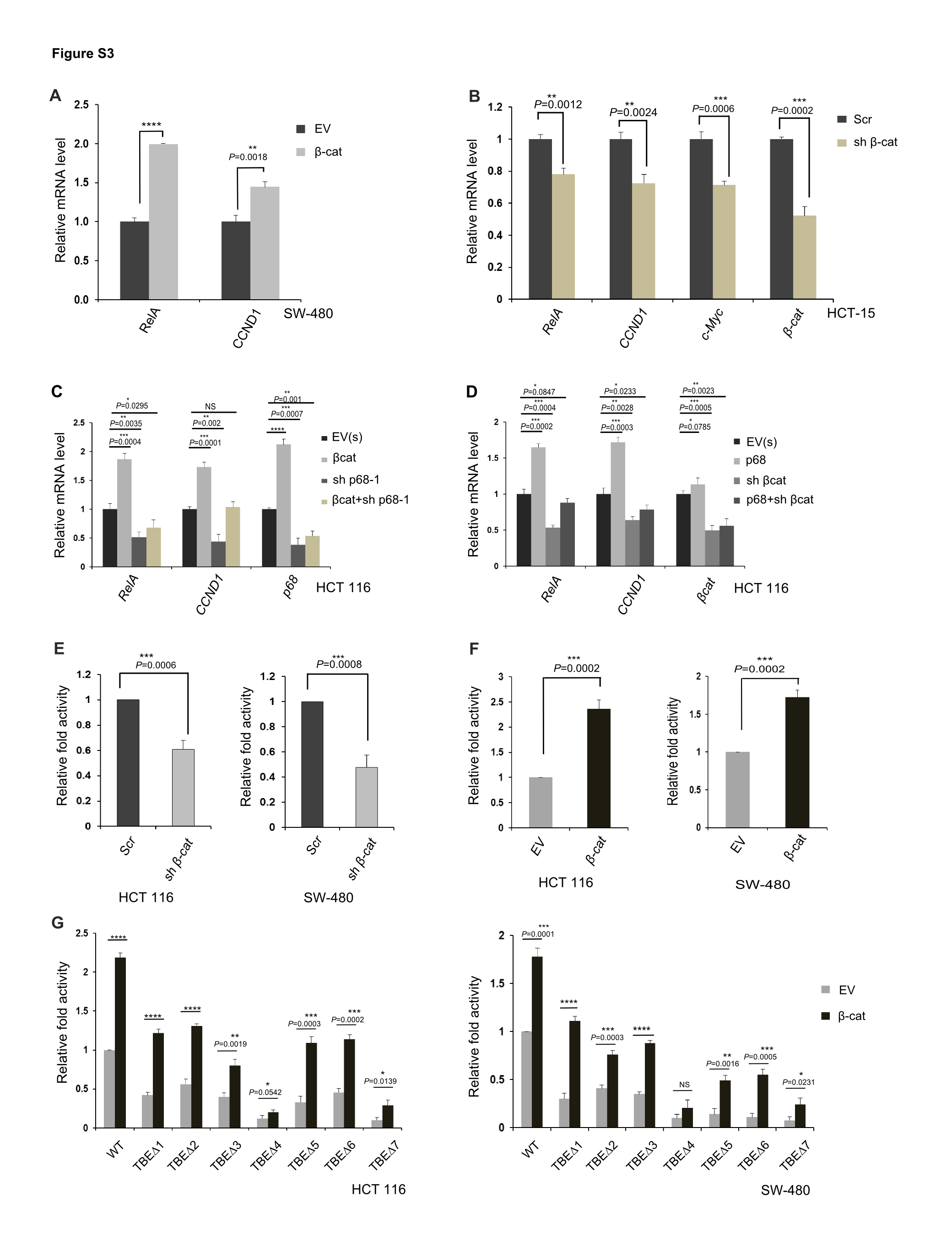

Supplement: Supplementary file 3 — Figure S3. Amalgamation between p68 and β-catenin regulates RelA expression. (A) SW-480 cells seeded in 35 mm plates were transfected with pGZ-β-catenin or EV (2 μg). (B) HCT-15 cells seeded in 35 mm plates were transfected with scr or β-catenin shRNA (2 μg). HCT 116 cells seeded in 35 mm plates were transfected with either (C) scr or p68 shRNA-1 (1 μg), in combination with pGZ-β-catenin or EV (1 μg) or (D) scr or β-catenin shRNA (1 μg), in combination with pGZ-p68 or EV (1 μg). Total RNA was extracted; 36 h post transfection for (A) and 48 h post transfection for (B), (C) and (D), followed by analysis of RelA mRNA by qRT–PCR. 18S rRNA was used for normalization in all the above experiments. HCT 116 (left) and SW-480 (right) cells seeded in 35 mm plates were transfected with (E) scr or β-catenin shRNA plasmid (1 μg) along with pGL3-RelA-prom (1 μg) and Renilla luciferase plasmid (50 ng). The luciferase activity was measured, 48 h post transfection. (F) EV or pGZ-β-catenin (1 μg) along with pGL3-RelA-prom (1 μg) and Renilla luciferase construct (50 ng). (G) pGZ-β-catenin or pGZ EV (1 μg) along with pGL3-WT-RelA-prom or its deletion constructs (1 μg) and Renilla luciferase plasmid (50 ng). For both (F) and (G) the luciferase activity was measured, 36 h post transfection. For (E), (F) and (G) cells co-transfected with (1 μg) of EV, pGL3-WT-RelA-prom and Renilla luciferase plasmid (50 ng). Renilla luciferase activity was used for normalization and data is represented as fold activity with respect to control cells. For all the sub-figures results are presented as mean (+) s.d. from three independent experiments. Indicated P-values were determined using Student’s t-test and P < 0.0001 is represented as ****. (TIF 407 kb) [file 13046_2019_1304_MOESM3_ESM.tif]

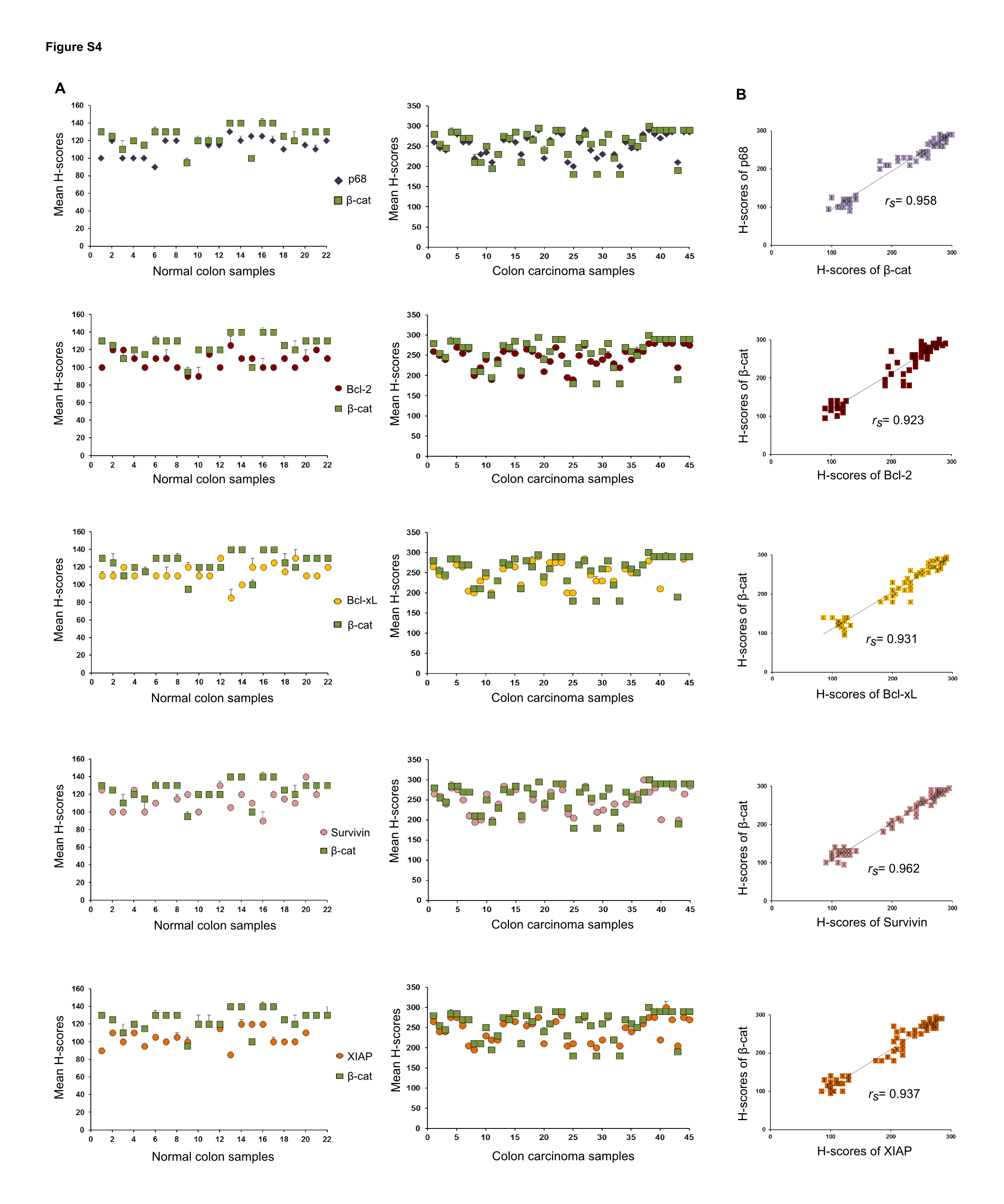

Supplement: Supplementary file 4 — Figure S4. Abundance of β-catenin with p68 and NF-κB target genes maintain strong positive correlation in human colon cancer tissues. (A) Scatter plots representing the mean H-scores of β-catenin and p68, Bcl-2, Bcl-xL, Survivin and XIAP in normal (n = 22) and colon carcinoma tissue (n = 45) samples, respectively. (B) Spearman’s rank correlation coefficient (rs) between the mean H-scores of β-catenin and p68, Bcl-2, Bcl-xL, Survivin and XIAP was determined from both normal and colon carcinoma tissues in combination. (TIF 628 kb) [file 13046_2019_1304_MOESM4_ESM.tif]
