# Supplementary material for: RNA helicase p68 deploys β-catenin in regulating RelA/p65 gene expression: implications in colon cancer
Source: J Exp Clin Cancer Res. 2019 Jul 27;38:330. doi: 10.1186/s13046-019-1304-y (PMC6660689; doi:10.1186/s13046-019-1304-y)
Supplement: Supplementary file 5 — Table S1. List of primers used in the study. (DOCX 19 kb) [file 13046_2019_1304_MOESM5_ESM.docx]

**Additional file 5**

**Table S1.** List of primers used in the study.

- ***Primer sequences used for cloning*:**

*RelA*-prom:

F-5’-AATAGATCTTGTGTAGGCATGCAGATATAAC-3’

R-5’-AATAAGCTTGGTGGGTCCGCCGATTAC-3’

- ***Primer sequences used for qRT-PCR*:**

*RelA*:

F-5’-ACAACAACCCCTTCCAAGTTCC-3’

R-5’-ACTGTCACCTGGAAGCAGAGC-3’

*Cyclin D1:*

F-5’-CCGTCCATGCGGAAGATC-3’

R-5’-GAAGACCTCCTCCTCGCACT-3’

*p68:*

F-5’-TGAGCGACCTTATCTCTGTGC-3’

R-5’-CCTGGAACGACCTGAACCTC-3’

*c-Myc:*

F-5’- CCAACAGGAACTATGACCTCGACTAC-3’

R-5’- CTCGAATTTCTTCCAGATATCCT-3’

*β-catenin:*

F-5’-TACCTCCCAAGTCCTGTATGAG-3’

R-5’-TGAGCAGCATCAAACTGTGTAG-3’

*18S rRNA:*

F-5’-GCTTAATTTGACTCAACACGGGC-3’

R-5’-AGCTATCAATCTGTCAATCCTGTC-3’

- ***Primers sequences used for deletion mutagenesis*:**

*RelA*-prom-TBE∆1:

F-5’-GGGCCCTGAAATCCCCTAAAAATGAGTAATCGGC-3’

R-5’-GCCGATTACTCATTTTTAGGGGATTTCAGGGCCC-3’

*RelA*-prom-TBE∆2:

F-5’-CAGTAGCCCTGGCTCCAGACCCGG-3’

R-5’-CCGGGTCTGGAGCCAGGGCTACTG-3’

*RelA*-prom-TBE∆3:

F-5’-GGCTGTAGGCTCCGCTCTTGATGCACTG-3’

R-5’-CAGTGCATCAAGAGCGGAGCCTACAGCC-3’

*RelA*-prom-TBE∆4:

F-5’-GTGGAGCATCCTCGGGATGAGGCCTG-3’

R-5’-CAGGCCTCATCCCGAGGATGCTCCAC-3’

*RelA*-prom-TBE∆5:

F-5’-GGTTCAATGATAATTGTATATCATTAACATTTTAATTAATCCATTCTGTCACCTGA-3’

R-5’-TCAGGTGACAGAATGGATTAATTAAAATGTTAATGATATACAATTATCATTGAACC-3’

*RelA*-prom-TBE∆6:

F-5’-CCTGTAATCCCAGCATGAGGCCAAGCGG-3’

R-5’-CCGCTTGGCCTCATGCTGGGATTACAGG-3’

*RelA*-prom-TBE∆7:

F-5’-GGCTCATGCCTGTAATCCCAGGAGGCTGAGG-3’

R-5’-CCTCAGCCTCCTGGGATTACAGGCATGAGCC-3’

- ***Primer sequences used for PCR in the ChIP Experiment*:**

*RelA TBE 1-3*-ChIP:

F-5’-TGTAGGCTCCGCAAAGCTCT-3’

R-5’-GGTCCGCCGATTACTCACTTTG-3’

*RelA TBE 4-ChIP:*

F-5’-AAACTGAAATGAGTGGGCGG-3’

R-5’- GACCCCACAGTGCAGGTAG-3’

*RelA TBE 5-ChIP:*

F-5’-TGGGTACATGGGTGTTTATCG-3’

R-5’-GACTTCAGGTGACAGAATGGATTA-3’

*RelA TBE 6-7-ChIP:*

F-5’-GCTCATGCCTGTAATCCCACTTT-3’

R-5’-CAACTTTCCTCCCCGCTTGG-3’

*Cyclin D1-ChIP:*

F-5’-GTAACGTCACACGGACTACAGG-3’

R-5’-GCACACATTTGAAGTAGGACACC-3’

*Actin-ChIP:*

F-5’- TGCACTGTGCGGCGAAGC-3’

R-5’- TCGAGCCATAAAAGGCAA-3’

- ***Primer sequences used for qRT-PCR in the ChIP Experiment*:**

*RelA TBE 1-3*-ChIP:

F-5’-TGTAGGCTCCGCAAAGCTCT-3’

R-5’-GGTCCGCCGATTACTCACTTTG-3’
